# Dipeptides in CSF and plasma: diagnostic and therapeutic potential in neurological diseases

**DOI:** 10.1007/s00726-024-03434-1

**Published:** 2024-12-13

**Authors:** Katharina Küper, Gernot Poschet, Julia Rossmann, Sven F. Garbade, Alexander Spiegelhalter, Dan Wen, Georg F. Hoffmann, Claus P. Schmitt, Thomas Opladen, Verena Peters

**Affiliations:** 1 Division of Pediatric Neurology and Metabolic Medicine, Department I, Medical Faculty Heidelberg, Center for Pediatric and Adolescent Medicine, Heidelberg University, Im Neuenheimer Feld 430, 69120 Heidelberg, Germany; 2 Centre for Organismal Studies (COS), Metabolomics Core Technology Platform, Heidelberg University, Heidelberg, Germany

**Keywords:** Dipeptide, CSF, Epilepsy

## Abstract

Dipeptides (DPs), composed of two amino acids (AAs), hold significant therapeutic potential but remain underexplored. Given the crucial role of AAs in central nervous system (CNS) function, this study investigated the presence of DPs in cerebrospinal fluid (CSF) and their correlation with corresponding AAs, potentially indicating their role as AA donors. Plasma and CSF samples were collected from 43 children with neurological or metabolic conditions of unknown origin, including 23 with epilepsy. A panel of 33 DPs was quantified using UPLC-MS/MS. Out of 33 DPs, 18 were detectable in CSF and 20 in plasma, displaying high inter-individual variance. Gly-Asp, Gly-Pro, and Ala-Glu were consistently found in all CSF samples, while only Gly-Asp was universally detectable in plasma. Anserine and carnosine were prominent in CSF and plasma, respectively, with no other histidine-containing DPs observed. Generally, DP concentrations were higher in plasma than in CSF; however, anserine and Gly-Pro had similar concentrations in both fluids. Significant correlations were observed between specific DPs and their corresponding AAs in CSF (Gly-Glu, Gly-Pro and Ser-Gln) and plasma (Glu-Glu and Glu-Ser). Notably, patients with epilepsy had elevated medium anserine concentrations in CSF. This study is the first to demonstrate the presence of numerous DPs in CSF and plasma. Further research is needed to determine if DP patterns can support the diagnosis of neurological diseases and whether DP administration can modulate amino acid availability in the brain, potentially offering new therapeutic options, such as for defects in the amino acid transporter.

**Supplementary Information:**

The online version contains supplementary material available at 10.1007/s00726-024-03434-1.

## Introduction

Amino acids (AAs) are fundamental to numerous metabolic pathways within the brain and crucial for maintaining neurological functions, while the role of dipeptides (DPs) on neurological functions remains unclear. DPs, comprised of two AAs, are products of protein degradation with diverse biological effects beyond serving as amino acid donors, such as induction of anti-oxidative mechanisms (Saadati 2023; Ozawa et al. 2022; Aldini et al. 2021), carbonyl sequestering activity (Aldini et al. 2021; Weigand et al. 2018), induction of H_2_S (Wetzel et al. 2023), interaction with glyceraldehyde 3-phosphate dehydrogenase (Moreno et al. 2021), exerts hydrolysis cleavage activity (Liu et al. 2018) or improvement of endothelial sealing (Bartosova et al. 2020). Further, non-proteinogenic amino acids have become a powerful tool for developing peptide-based drug candidates (Ding et al. 2020).

Imbalances in AA metabolism, often caused by abnormalities in AA transporters, have been linked to neurological disorders, including epilepsy (Traynelis et al. 2010; Lee 2011; Malik and Willnow 2019), schizophrenia (Adla et al. 2024), autism spectrum disorders (Brister et al. 2022) or Alzheimer’s disease (Nuzzo et al. 2021). Central to the pathophysiology is glutamate, the primary excitatory neurotransmitter in the cerebrospinal fluid (CSF), which serves as a precursor for gamma-aminobutyric acid (GABA), a key inhibitory neurotransmitter synthesized by l-glutamate decarboxylase (Bryson et al. 2023; Albrecht et al. 2007). The glutamate-glutamine cycle, a cyclical metabolic pathway, plays a pivotal role in maintaining a sufficient supply of the glutamate in the central nervous system. Notably, neurons cannot synthesize either glutamate or GABA directly from glucose. Furthermore, beyond the glutamate-glutamine axis, several other amino acids such as aspartate, glycine, GABA, tryptophan, histidine and tyrosine, pivotal for neurotransmission or as precursors to neurotransmitters, are essential for normal neurological function. Recent studies have shed light on alterations in the concentrations of various amino acids, including beta-alanine, glycine, serine, arginine, proline, methionine, cysteine and alanine in the cerebrospinal fluid (CSF) of patients with status epilepticus (Hanin 2024). However, the role of DPs in the brain remains largely unclear. In patients with schizophrenia, not only a reduced concentration of glutamate but also of DP γ-Glu-Gln was observed in the CSF (Do et al. 1995). Even though it has been shown that the administration of carnosine plays a protective role in neurological diseases, it is still unclear to what extent oral administration of carnosine can influence CSF levels (Hegazy et al. 2022).

The aim of this study is to investigate for the first time whether DPs are present in CSF and whether they can potentially serve as AA donors. In case of proteins that only occur in low concentrations in the CSF, increases are considered to be signs of inflammation, bleeding or other pathologies. Using ultra-performance liquid chromatography (UPLC) coupled with tandem mass spectrometry (Heidenreich et al. 2021), we attempt to identify DPs and AAs in epilepsy patient CSF and plasma collected simultaneously from patients. Because collecting CSF samples from healthy children is difficult, most CSF samples are from paediatric patients with neurological symptoms. This investigation holds promise for the discovery of new biomarkers and therapeutic targets in neurological disorders related to abnormalities in AA concentrations.

## Materials and methods

### Plasma and CSF samples

All patients have been admitted to Heidelberg University Hospital for further evaluation of unexplained neurological and/or metabolic conditions, including 23 patients with epilepsy or a history of seizures. Plasma and CSF was collected from patients taken at the same time. The children were aged between 2 weeks and 17 years (17 females and 26 males). Since the DP concentrations in cerebrospinal fluid and plasma are not very stable due to the presence of endogenous peptidases, all samples were immediately frozen after collecting and stored at − 80°C. Epilepsy was classified into "epilepsy yes or no" and "focal" or "generalized". All samples were acquired between January and March 2023. This study was approved by the ethic committee of the University Heidelberg (S-554/2018).

### Dipeptide and amino acid quantification

33 DP were determined by an UPLC-MS/MS method as described by Heidenreich (Heidenreich, et al. 2021). Alanyl-Alanine (Ala-Ala), Alanyl-Glutamine (Ala-Gln), Alanyl-Glutamate (Ala-Glu), Alanyl-Glycine (Ala-Gly), Alanyl-Histidine (Ala-His), Alanyl-Phenylalanine (Ala-Phe), Alanyl-Proline (Ala-Pro), Alanyl-Tyrosine (Ala-Tyr), Anserine, Aspartyl-Glutamine (Asp-Gln), Carnosine, Glutamyl-Glutamate (Glu-Glu), γ-Glutamyl-ε-Lysine (γ-Glu-ε-Lys), Glutamyl-Serine (Glu-Ser), Glycyl-Aspartate (Gly-Asp), Glycyl-Glutamate (Gly-Glu), Glycyl-Histidine (Gly-His), Glycyl-Phenylalanine (Gly-Phe), Glycyl-Proline (Gly-Pro), Histidyl-Alanine (His-Ala), Histidyl-Leucine (His-Leu), Histidyl-Serine (His-Ser), Leucyl-Histidine (Leu-His), Leucyl-Proline (Leu-Pro), Phenylalanyl-Alanine (Phe-Ala), Prolyl-Glycine (Pro-Gly), Prolyl-Leucine (Pro-Leu), Seryl-Alanine (Ser-Ala), Seryl-Glutamine (Ser-Gln), Seryl-Histidine (Ser-His), Tyrosyl-Alanine (Tyr-Ala), Tyrosyl-Phenylalanine (Tyr-Phe), Valyl-Tyrosine (Val-Tyr) were measured via UPLC-MS/MS. A panel of 18 amino acids were determined as previously described by Weger et al. (2016) including alanine (Ala), arginine (Arg), asparagine (Asn), aspartate (Asp), glutamine (Gln), glutamate (Glu), glycine (Gly), histidine (His), isoleucine (Ile), leucine (Leu), lysine (Lys), methionine (Met), phenylalanine (Phe), proline (Pro), serine (Ser), threonine (Thr), tyrosine (Tyr) and valine (Val).

### Method validation

The method has been established and validated as described previously (Heidenreich et al. 2021). In brief, each DP is represented by a specific multiple reaction monitoring with the fragment mass 171.1, derived from the AccQ-Tag™ reagent. A second qualifying fragment (Q3) was included where possible. MS/MS parameters were optimized for all derivatized DPs via manual tuning and flow-injection analysis. Baseline separation was achieved for isomeric DPs. Ala-Leu and Leu-Ala shared the same retention time and could not be discriminated. Cys-Gly had a high background peak, affecting quantification. Gly-Leu and Leu-Gly separated well but showed a strong background peak, complicating quantification at lower concentrations. Signal intensity and calibration range vary between DPs. The limit of quantification ranged from 0.1 to 2.5 fmol depending on the analyte. DPss with multiple amine groups, like His-containing DPs, generally showed lower signal intensities compared to those with a single amine group.

### Statistical analysis

Results in figures are presented as mean ± SD unless stated otherwise. Correlations were calculated using Spearman correlation coefficient via Graph Pad Prism. Mann-Whitney test and Area-under-the-curve analysis were used for determination of a statistically significant difference between two groups via Graph Pad Prism. Age- and gender-specific effects have been evaluated by multiple linear regression analysis.

## Results

### Dipeptide distribution in plasma and CSF

Out of the 33 DPs investigated, 18 were detectable in CSF and 20 in plasma, with Gly-Asp, Gly-Pro and Ala-Glu present in all 43 CSF samples, and only Gly-Asp in all plasma samples. Ala-Pro, Ala-Gly, Ala-Phe were only detectable in plasma but not in CSF, Leu-Pro was only detectable in CSF and not in plasma. DPs that could not be determined due to low detection (below baseline), due to a very poor signal-to-noise ratio or strong overlap with an unknown co-eluting peak have not been considered in the calculations.

**Fig. 1:**
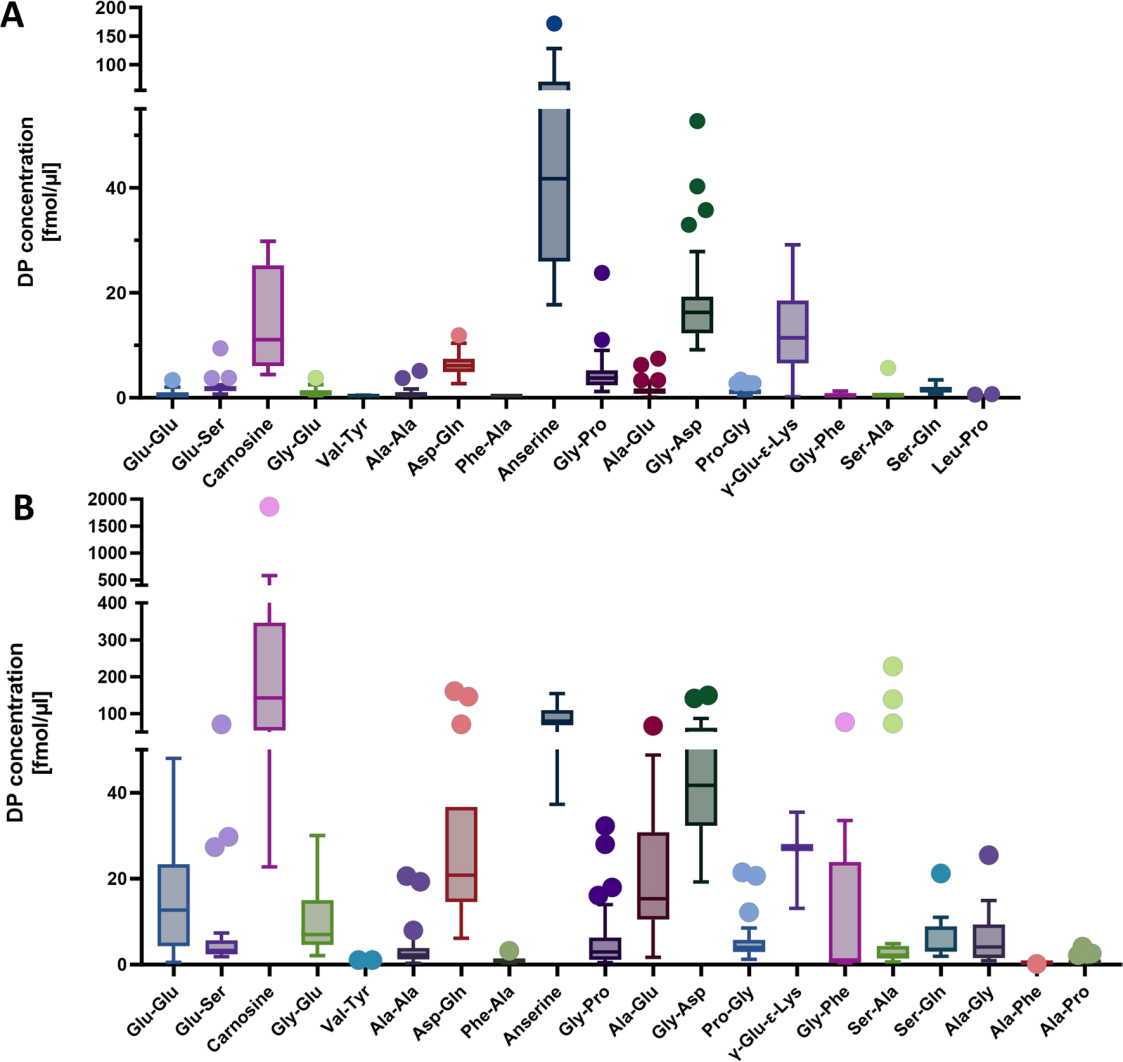
Dipeptide concentrations (fmol/µl) in CSF (**A**) and plasma (**B**) of 43 CSF samples from children with neurological or metabolic conditions of unknown origin. In CSF, out of 43 patients, anserine was detectably in 24 patients, Gly-Asp in 43, Carnosine in 4, γ-Glu-ε-Lys in 36, Asp-Gln in 26, Gly-Pro in 43, Glu-Ser in 21, Ser-Gln in 17, Ala-Glu in 43, Pro-Gly in 42, Gly-Glu in 23, Ala-Ala in 24, Ser-Ala in 13, Glu-Glu in 21, Gly-Phe in 3, Val-Tyr in 22, Phe-Ala in 3 and Leu-Pro in 13. In plasma, anserine was detectably in 12 patients, Gly-Asp in 43, Carnosine in 12, γ-Glu-ε-Lys in 3, Asp-Gln in 14, Gly-Pro in 42, Glu-Ser in 25, Ser-Gln in 17, Ala-Glu in 42, Pro-Gly in 39, Gly-Glu in 37, Ala-Ala in 28, Ser-Ala in 23, Glu-Glu in 37, Gly-Phe in 9, Val-Tyr in 21, Phe-Ala in 15, Ala-Gly in 27, Ala-Pro in 24 and Ala-Phe in 1. Displayed is a Tukey-Box-Plot

The histidine-containing DP anserine was the highest occurring DP in the CSF, followed by Gly-Asp, carnosine and *γ-*Glu*-ε-*Lys. In plasma, carnosine was the DP with the highest mean concentration, albeit only in 12 of 43 plasma samples detectable, followed by anserine (Fig. 1). None of the other histidine-containing DPs measured were detectable in plasma and CSF (Ala-His, His-Ala, His-Leu, His-Ser, Leu-His, Ser-His). Since Glu and Gln play a central role in neurotransmission in brain, we investigated how many of the DPs contain these two amino acids. All 4 investigated Glu-containing DPs (Glu-Ser, Ala-Glu, Gly-Glu, Glu-Glu) were detectable and 2 (Asp-Gln and Ser-Gln) out of 3 Gln-containing DPs were detectable in the CSF samples, Ala-Gln was below detection limit. Other DPs containing AAs involved in neurotransmitters, such as aspartate or glycine, were also present in the CSF (Asp-Gln, Gly-Asp, Gly-Pro, Pro-Gly, Gly-Glu, Gly-Phe). The multiple linear regression analysis did not reveal any gender- or age-specific distribution patterns for the DPs in CSF and plasma, with the exception of anserine in CSF, which was found to be reduced in patients under 10 years of age (p < 0.03). However, due to the limited sample size, particularly among patients older than 10 years,drawing definitive conclusions is challenging.

### Correlation analysis of dipeptides and amino acids in plasma and CSF

To investigate whether the DP concentration is related to the AA concentration, we determined the correlations between the DPs present in CSF and plasma (n = 15) and their respective AAs in C- or N-terminus. In CSF, Gly-Glu, Gly-Pro, Ser-Gln correlated with both corresponding AAs while Ala-Glu, Gly-Asp and Pro-Gly only correlated with one of their respective AAs (r > 0.5 and p < 0.05). No correlation was found for Ala-Ala, Ser-Ala, Glu-Glu, Glu-Ser, Val-Tyr, Asp-Gln, Phe-Ala, Leu-Pro and Gly-Phe with their respective AAs (Table 1).

**Table 1:** Correlation of DPs in CSF with their respective AAs at N-terminal and C-terminal (Spearman)

DP	Correlation (r) N-terminal	Significance (p)	Number of patients	Correlation (r) C-terminal	Significance (p)	Number of patients
Gly-Glu	0.68	< 0.01	22	0.57	< 0.01	21
Gly-Pro	0.55	< 0.01	42	0.68	< 0.01	33
Ser-Gln	0.54	0.03	17	0.64	< 0.01	17
Ala-Glu	0.50	< 0.01	42	0.24	0.23	40
Gly-Asp	0.51	< 0.01	42	0.34	0.30	11
Pro-Gly	0.21	0.23	33	0.50	< 0.01	42
Ala-Ala	− 0.18	0.39	25	− 0.18	0.39	25
Ser-Ala	− 0.03	0.92	13	− 0.05	0.88	13
Glu-Glu	0.29	0.22	19	0.29	0.22	19
Glu-Ser	0.46	0.04	20	0.36	0.11	21
Val-Tyr	0.43	0.04	22	0.30	0.18	22
Asp-Gln	0.36	0.43	7	0.43	0.02	26
Leu-Pro	0.30	0.32	13	− 0.05	0.89	11

In plasma, Glu-Glu and Glu-Ser correlated with their respective N- and C-terminal AAs. Asp-Gln, Ala-Glu, Pro-Gly, Ser-Ala and Gly-Pro correlated with one of their respective AAs (r > 0.5 and p < 0.05). No correlation was found for Ala-Ala, Ala-Pro, Gly-Glu, Ala-Gly, Gly-Asp, Ser-Gln, Val-Tyr, Phe-Ala and Gly-Phe with their respective AAs. More DPs (n = 6) correlated with their N-terminal AA than with the C-terminal AA (n = 3, Suppl. Table 1).

### Comparative analysis of dipeptide and amino acid profiles in plasma and CSF

To understand the relationship between plasma and CSF DP profile, we determined the relation of DP and AA concentrations between plasma and CSF. 17 of the DPs and 18 AAs were detectable in both, CSF and plasma (Table 2, Suppl. Table 2). While the concentrations of some DPs in CSF and plasma were in a similar concentration range, such as anserine or Gly-Pro, the concentration of other DPs, such as Ser-Ala or Gly-Phe, was many times lower in CSF than in plasma. Even though the concentration of Pro-Gly and Gly-Pro in plasma were in a similar range, the concentration of Pro-Gly in CSF was much lower than that of its stereoisomer. The concentrations of proline and glutamate were much lower in CSF than in plasma (Suppl. Table 2). A significant correlation between CSF and plasma DP concentrations was found for Ala-Ala (r > 0.78; p < 0.01; Suppl Table 3).

**Table 2:** Mean concentrations of DPs in plasma and CSF and their ratio

DP	Plasma Mean (fmol/µl) ± SD	CSF Mean (fmol/µl) ± SD	Ratio Plasma/CSF per patient Mean ± SD	Number of patients
Carnosine	325.05 ± 487.58	14.10 ± 9.47	25.04 ± 26.62	3
Anserine	85.83 ± 32.22	55.63 ± 38.50	2.55 ± 1.73	11
Gly-Asp	49.99 ± 27.26	17.86 ± 8.68	2.94 ± 1.15	43
Asp-Gln	40.51 ± 48.46	6.33 ± 2.13	6.12 ± 5.04	7
γ-Glu-ε-Lys	25.28 ± 9.22	12.56 ± 7.01	2.90 ± 0.68	2
Ala-Glu	21.47 ± 14.91	1.55 ± 1.41	23.58 ± 30.59	42
Ser-Ala	21.20 ± 53.82	0.76 ± 1.43	152.91 ± 213.91	6
Glu-Glu	16.29 ± 13.38	0.70 ± 0.77	43.47 ± 49.46	16
Gly-Phe	14.28 ± 24.41	0.51 ± 0.54	497.73 ± 497.55	2
Gly-Glu	9.49 ± 6.37	1.14 ± 0.76	11.11 ± 6.27	22
Glu-Ser	8.20 ± 14.55	2.17 ± 1.79	4.49 ± 5.39	12
Ser-Gln	6.13 ± 5.47	1.64 ± 0.72	5.39 ± 4.99	3
Gly-Pro	5.65 ± 7.03	4.48 ± 3.69	1.31 ± 1.37	42
Pro-Gly	5.19 ± 4.21	1.30 ± 0.74	4.86 ± 4.67	38
Ala-Ala	3.83 ± 4.82	0.90 ± 1.15	5.66 ± 3.25	17
Phe-Ala	0.97 ± 0.82	0.07 ± 0.06	6.72 ± 0.00	1
Val-Tyr	0.38 ± 0.32	0.15 ± 0.13	6.48 ± 11.00	11

Mean of the ratios of DPs between plasma and CSF individually for each patient

### Dipeptide levels in CSF of children with and without epilepsy: a comparative study

**Fig. 2:**
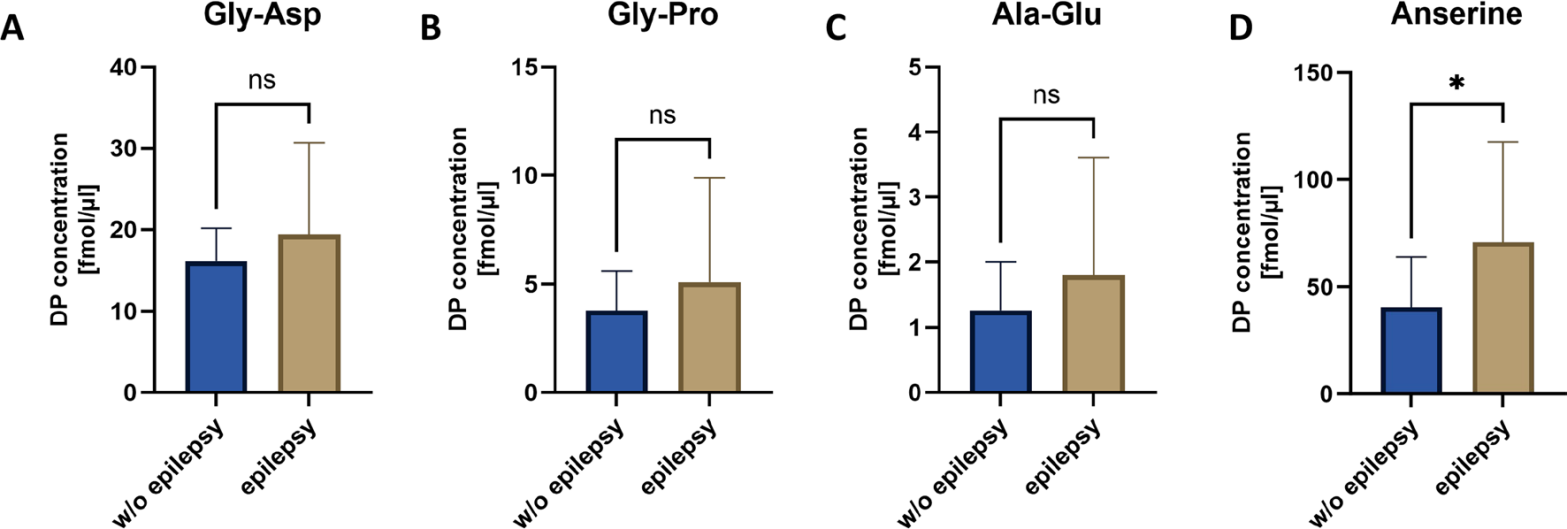
The concentrations of the three DPs present in all patients, Gly-Asp (**A**), Gly-Pro (**B**) and Ala-Glu (**C**) and anserine (**D**), the DP with the highest concentration on average in CSF of patients without (w/o) and with epilepsy (in fmol/µl). Anserine was detectable in 12 patients without and with epilepsy, Gly-Asp, Gly-Pro and Ala-Glu in 20 patients without and in 23 patients with epilepsy. The mean values of these DPs tend to be higher for patients with epilepsy than for patients without epilepsy, but did only reach statistical significance in anserine. Mann–Whitney test was used for statistical evaluation. Displayed is mean ± SD. *p < 0.05. ns = not significant

We compared DP/AA concentrations in children with and without epilepsy. Out of the 23 patients with epilepsy, 4 had focal seizure, while 10 were classified as having generalized epilepsy. For 9 patients, information on the type of seizure was missing. Three of the patients received valproate (VPA), one with focal and one with generalized epilepsy (Suppl. Table 4). There was no obvious difference in DP and AA pattern in CSF of the patients receiving valproate. The DP concentrations varied greatly between patients. The mean concentrations of the three DPs that were detectable in all CSF samples (Gly-Asp, Gly-Pro, Ala-Glu) tend to be higher in patients with epilepsy than in patients without epilepsy, but without reaching statistical significance (Fig. 2). Anserine, the DP present at the highest average concentration, was found to be 1.8 times higher in patients with epilepsy compared to those without epilepsy (p < 0.05). However, this difference may be influenced by age, as the epilepsy group was, on average, younger than the non-epilepsy group (3 years vs. 7 years, respectively). This observation was further supported by area-under-the-curve (AUC) analysis, which showed the following AUC values for Gly-Asp, Gly-Pro, and Ala-Glu: 0.504, 0.554, and 0.555, respectively (Confidence Intervals: 0.35–0.68, 0.77–0.73, and 0.37–0.72, p = n.s.). In contrast, only for anserine the AUC was significantly higher (AUC: 0.75; Confidence Interval: 0.55–0.95; p < 0.037). There was no detectable difference in DPs between the small sub-cohorts of patients with generalized and focal seizures in CSF and plasma (Suppl. Table 5 A-D).

## Discussion

Dipeptides (DPs) represent a class of compounds that have received limited attention in scientific research and human biology until now. DPs can originate from both dietary intake and internal protein breakdown, with the potential for 400 different DPs to emerge from the 20 proteinogenic Amino acids (AAs). Recent investigations have unveiled a significant presence of DPs across various organs, urine, and serum of mice (Heidenreich et al. 2021), as well as in the human kidney (Pfeffer et al. 2024). Beyond serving as AA donors they have diverse biological effects such as induction of anti-oxidative mechanisms (Saadati 2023; Ozawa et al. 2022; Aldini et al. 2021), carbonyl sequestering activity (Aldini et al. 2021; Weigand et al. 2018), induction of H_2_S (Wetzel et al. 2023), interaction with glyceraldehyde 3-phosphate dehydrogenase (Moreno et al. 2021) or improvement of endothelial sealing (Bartosova et al. 2020). Notably, DPs exhibit distinct distribution patterns among organs in mice, hinting at an organ-specific metabolic regulation within the body. Recognizing the pivotal role of AAs in neurological functions, we aimed to explore the presence of DPs in the cerebrospinal fluid (CSF), their correlation with corresponding AAs and the potential relationship between plasma and CSF DP profiles.

We demonstrate the presence of numerous DPs in both CSF and plasma in humans, albeit at relatively low concentrations. The DP concentration in CSF and plasma was significantly lower than that of the AAs, but if one considers the sum of all 400 DPs, the concentrations of all DPs are presumably in a similar concentration range as their corresponding AAs. In particular, the inter-individual differences in DP concentrations were considerable, as only certain DPs were detected in all patients. Among the detected DPs, anserine emerged with notably higher concentrations than carnosine and other DPs, while other histidine-containing DPs, apart from anserine and carnosine, were absent. The presence of carnosine and anserine in the brain has already been demonstrated in humans (Boldyrev et al. 2013). Anserine is formed by the methylation of carnosine (Kwiatkowski et al. 2018) and both DPs are degraded by the Carnosinase 1 (CN1), an enzyme which very specifically degrades only histidine-containing DPs, also present in the brain (Teufel et al. 2003). A disorder of carnosinase 1, carnosinemia or homocarnosinemia, has been described, but a connection between the enzyme and the phenotype has never been shown (Sjaastad et al. 2018; Peters et al. 2018; Pfeffer et al. 2023). In contrast, numerous therapeutic approaches are being investigated for these DPs (Caruso et al. 2023), e.g. in the context of cognitive functions and their decline (Caruso et al. 2021; Masuoka et al. 2021), neurodegenerative diseases (Solana-Manrique et al. 2022), cancer (Maugeri et al. 2023), late complications of diabetes (Matthews et al. 2021; Peters et al. 2018) in humans or in an epilepsy rat model (Qi et al. 2018). Furthermore, Gly-Asp was detectable at relatively high levels, consistent with its prevalence in various mouse organs, including the brain (Heidenreich et al. 2021). Gly-Asp cannot be degraded by CN1 or CN2 (Pfeffer et al. 2024). It is an anionic DP with a high resistance to hydrolysis compared to DPs with bulky residues in the N-terminal position (Rohm et al. 2019a, b). Additionally, DPs containing Glu, Gln, Asp, and Gly residues were prominently present in the CSF of the patients.

In line with previous studies (Akiyama et al. 2014), the AA concentrations were higher in plasma than in CSF, especially for proline and glutamate. While the concentrations for some DPs were in the same range in plasma and CSF, e.g. for anserine or Gly-Pro, the concentrations of Ser-Ala and Gly-Phe in CSF were many times lower than in plasma. The plasma/CSF ratio for AAs is considered as an indicator of AA transport with several transporters described (Zaragozá 2020). Cellular uptake of DPs occurs by proton-coupled oligopeptide transporters (POTs) via an inwardly-directed proton gradient and negative membrane potential. At present, four members of the POT family, namely PEPT1 (SLC15A1), PEPT2 (SLC15A2), PHT1 (SLC15A4) and PHT2 (SLC15A3), have been identified in mammals (Jappar et al. 2009). In the brain, PEPT2 in particular seems to play an important role contributing to the peptide homeostasis (Smith et al. 2013). To date, the transport of DPs has primarily been investigated during intestinal absorption and little is known about transport across the blood–brain barrier. The intestinal uptake of DPs and their distribution in the blood depends very much on the DP structure (Rohm et al. 2019a, b). However, in a previous study the DPs glycyl-glutamine and glycyl-tyrosine were administered to healthy individuals and their concentrations were quantified in blood and CSF. However, the administration of these DPs did not increased their concentration in the CSF (Himmelseher et al. 1996). Whether other DP can pass the blood–brain barrier may depend on their structure and needs further studies. These DPs could be of therapeutic use, both in their function as AA donors and in their properties that go beyond those of the individual AAs. The correlation of DPs between plasma and CSF is an important parameter to better understand an indication of a possible transport of the DP into the CSF. Although the correlations between plasma and CSF concentrations were rather low in our cohort, a correlation for some of the DPs was detected, especially alanine and glycine-containing DPs. Considering the role of DPs as AA donors, we tested the correlation between DPs and their corresponding AAs. In CSF, our cohort shows for several DPs a correlation with their corresponding AAs. However, DPs are not the only source of AAs. Additionally, previous findings from DP and AA profiles in different tissues of mice suggest the influence of metabolic activity on their relation (Heidenreich et al. 2021). Even though the influence of valproate on AA concentrations in the brain has been proven (Wisłowska-Stanek et al. 2024; Anderson et al. 1994), we could not find a clear influence of valproate on the DP and AA pattern in our cohort. Studies in larger cohorts would be necessary for a clear statement.

The present study shows for the first time the presence of many DPs in blood and CSF. This study is a preliminary study with some limitations. Although there was a trend that DP concentrations in epileptic children were generally higher as for non-epileptic children but this trend reached significant differences only for anserine, which might be caused by age-dependent effects. However, there were no clear indications of sex- or age-dependent variability of the DPs, except anserine, in plasma and CSF, but more samples are needed for definitive conclusions. The DP concentration also shows a high intra-individual range, so this trend should be viewed with caution. As it is generally difficult to obtain plasma and CSF samples from children, the group size of 43 patients is limiting for statistical conclusions. In addition, we only used samples from which plasma and CSF samples were taken simultaneously. Since healthy children do not undergo CSF and plasma sampling, the controls are also children with unexplained neurological symptoms. This is another factor that makes it difficult to compare the patient groups. The influence of food on DP concentrations was also not considered in this study. Even if the concentrations of AAs and DPs in the serum are strongly dependent on the diet and certainly explain part of the strong inter-individual differences, the ratio of DPs in serum and CSF is certainly only minimally influenced by this. Additionally, the DPs and AAs are components of a continuous metabolic process. The measurements can, of course, only provide a snapshot of this process. Despite these limitations, this study reveals new, previously undescribed findings on DPs and their corresponding AAs and will hopefully stimulate further investigations.

In conclusion, this study is the first to identify the presence of numerous DPs in cerebrospinal fluid (CSF). Measuring DP concentrations can indicate pathological changes. For example, we found that mean anserine concentrations, the DP with the highest concentration in CSF, are elevated in patients with epilepsy. Since several detected DPs in CSF correlate with their respective AAs and some with their plasma concentrations, therapeutic concepts involving the administration of DPs for neurological diseases with altered AA levels are conceivable. While therapeutic approaches targeting DPs are being explored for various diseases, further research is needed to clarify their exact functions and effects on neurological conditions.

## Supplementary Information

Below is the link to the electronic supplementary material.

Supplementary file1 (DOCX 169 KB)

## Data Availability

No datasets were generated or analysed during the current study.
