# Supplementary material for: Dipeptides in CSF and plasma: diagnostic and therapeutic potential in neurological diseases
Source: Amino Acids. 2024 Dec 13;57:2. doi: 10.1007/s00726-024-03434-1 (PMC11645304; doi:10.1007/s00726-024-03434-1)
Supplement: Supplementary file 1 [file 726_2024_3434_MOESM1_ESM.docx]

*Supplement*

**Suppl. Table 1: Correlation of DPs in plasma with their respective AAs at the N- and C-terminal (using Spearman correlation coefficient)**

| DP | Correlation (r)  N-terminal | p | Number of patients | Correlation (r)  C-terminal | p | Number of patients |
| --- | --- | --- | --- | --- | --- | --- |
| Glu-Glu | 0.77 | <0.01 | 37 | 0.77 | <0.01 | 37 |
| Glu-Ser | 0.50 | 0.01 | 25 | 0.56 | <0.01 | 25 |
| Asp-Gln | 0.75 | <0.01 | 14 | 0.31 | 0.27 | 14 |
| Ala-Glu | 0.55 | <0.01 | 42 | 0.43 | <0.01 | 42 |
| Pro-Gly | 0.54 | <0.01 | 39 | 0.14 | 0.40 | 39 |
| Ser-Ala | 0.53 | 0.01 | 23 | 0.15 | 0.49 | 23 |
| Gly-Pro | 0.33 | 0.03 | 42 | 0.64 | <0.01 | 42 |
| Ala-Ala | -0.10 | 0.61 | 28 | -0.10 | 0.61 | 28 |
| Ala-Pro | -0.01 | 0.95 | 24 | 0.24 | 0.26 | 24 |
| Gly-Glu | 0.36 | 0.03 | 37 | 0.37 | 0.02 | 37 |
| Ala-Gly | 0.15 | 0.46 | 27 | 0.42 | 0.03 | 27 |
| Gly-Asp | 0.19 | 0.23 | 43 | 0.43 | <0.01 | 43 |
| Ser-Gln | 0.32 | 0.34 | 11 | 0.46 | 0.16 | 11 |
| Val-Tyr | 0.45 | 0.04 | 21 | 0.44 | 0.05 | 21 |
| Phe-Ala | 0.38 | 0.17 | 15 | -0.03 | 0.93 | 15 |
| Gly-Phe | 0.50 | 0.18 | 9 | 0.40 | 0.29 | 9 |

**Suppl. Table 2: Mean concentrations of AAs in plasma and CSF and their ratio**

| AA | Plasma  Mean (pmol/µl) ± SD | CSF  Mean (pmol/µl) ± SD | Ratio Plasma/CSF per patient  Mean ± SD | Number of patients |
| --- | --- | --- | --- | --- |
| Glutamine | 600.06 ± 119.40 | 261.30 ± 60.13 | 6.26 ± 25.73 | 43 |
| Alanine | 325.42 ± 106.01 | 25.92 ± 10.91 | 13.60 ± 4.16 | 42 |
| Glycine | 297.04 ± 77.15 | 21.29 ± 7.78 | 15.17 ± 4.22 | 42 |
| Valine | 241.82 ± 65.88 | 17.23 ± 8.06 | 15.55 ± 5.01 | 43 |
| Proline | 222.98 ± 93.76 | 1.43 ± 2.20 | 1209.96 ± 2703.43 | 33 |
| Serine | 160.53 ± 40.10 | 34.42 ± 14.98 | 5.10 ± 1.56 | 42 |
| Lysine | 152.67 ± 60.58 | 20.39 ± 7.36 | 7.67 ± 1.97 | 43 |
| Leucine | 144.54 ± 51.44 | 13.40 ± 6.96 | 11.98 ± 3.86 | 43 |
| Threonine | 135.65 ± 48.65 | 26.99 ± 12.93 | 5.45 ± 1.43 | 43 |
| Glutamate | 90.00 ± 41.53 | 0.77 ± 1.08 | 452.55 ± 681.90 | 40 |
| Phenylalanine | 87.78 ± 91.26 | 13.09 ± 21.69 | 8.44 ± 2.59 | 43 |
| Isoleucine | 83.34 ± 37.07 | 5.58 ± 3.56 | 17.45 ± 6.77 | 43 |
| Histidine | 75.83 ± 16.58 | 11.76 ± 5.93 | 7.29 ± 2.28 | 43 |
| Tyrosine | 72.03 ± 26.59 | 10.18 ± 5.48 | 7.86 ± 2.92 | 43 |
| Arginine | 70.54 ± 29.89 | 16.54 ± 6.64 | 4.45 ± 1.50 | 43 |
| Asparagine | 57.82 ± 17.89 | 6.74 ± 3.25 | 9.56 ± 3.15 | 43 |
| Methionine | 25.45 ± 12.16 | 2.43 ± 1.91 | 14.70 ± 8.57 | 43 |
| Aspartate | 11.19 ± 10.44 | 1.96 ± 3.88 | 32.51 ± 38.57 | 11 |

**Suppl. Table 3: Correlation of DPs in CSF with DPs in plasma (using Spearman correlation coefficient)**

| DP | Correlation (r) CSF/Plasma | *p* | Number of patients |
| --- | --- | --- | --- |
| Ala-Ala | 0.78 | <0.01 | 17 |
| Gly-Glu | 0.48 | 0.03 | 22 |
| Gly-Pro | 0.47 | <0.01 | 42 |
| Gly-Asp | 0.41 | 0.01 | 43 |
| Ala-Glu | 0.32 | 0.04 | 42 |
| Pro-Gly | 0.08 | 0.64 | 38 |
| Glu-Ser | 0.49 | 0.11 | 12 |
| Ser-Ala | -1.00 | 0.30 | 6 |
| Carnosine | 0.50 | 1.00 | 3 |
| Asp-Gln | 0.50 | 0.27 | 7 |
| Ser-Gln | 0.11 | 1.00 | 3 |
| Glu-Glu | -0.15 | 0.57 | 16 |
| Val-Tyr | 0.46 | 0.16 | 11 |
| Anserine | -0.08 | 0.82 | 11 |

**Suppl Table 4: Clinical information of the 43 patients**

| patient | Birth year | sex  [male/female = m/f] | Epilepsy | Valproate | Type of epilepsy  [focal/generalized/  unclear/no epilepsy] |
| --- | --- | --- | --- | --- | --- |
| 1 | 2019 | m | yes | ? | focal |
| 2 | 2022 | m | yes | no | focal |
| 3 | 2021 | f | yes | no | focal |
| 4 | 2019 | f | yes | yes | focal |
| 5 | 2022 | m | yes | ? | generalized |
| 6 | 2022 | m | yes | no | generalized |
| 7 | 2009 | m | yes | no | generalized |
| 8 | 2022 | f | yes | no | generalized |
| 9 | 2022 | f | yes | no | generalized |
| 10 | 2022 | f | yes | no | generalized |
| 11 | 2021 | f | yes | no | generalized |
| 12 | 2020 | m | yes | yes | generalized |
| 13 | 2022 | m | yes | no | generalized |
| 14 | 2020 | m | yes | no | generalized |
| 15 | 2022 | f | yes | no | unclear |
| 16 | 2020 | m | yes | no | unclear |
| 17 | 2022 | m | yes | ? | unclear |
| 18 | 2023 | f | yes | no | unclear |
| 19 | 2017 | f | yes | ? | unclear |
| 20 | 2012 | m | yes | no | unclear |
| 21 | 2006 | m | yes | no | unclear |
| 22 | 2021 | m | yes | no | unclear |
| 23 | 2007 | m | yes | yes | unclear |
| 24 | 2021 | m | no | no | no epilepsy |
| 25 | 2006 | f | no | no | no epilepsy |
| 26 | 2020 | f | no | no | no epilepsy |
| 27 | 2008 | f | no | no | no epilepsy |
| 28 | 2017 | m | no | no | no epilepsy |
| 29 | 2009 | m | no | no | no epilepsy |
| 30 | 2018 | m | no | no | no epilepsy |
| 31 | 2023 | m | no | no | no epilepsy |
| 32 | 2020 | m | no | no | no epilepsy |
| 33 | 2007 | f | no | no | no epilepsy |
| 34 | 2020 | f | no | no | no epilepsy |
| 35 | 2016 | m | no | ? | no epilepsy |
| 36 | 2021 | f | no | no | no epilepsy |
| 37 | 2019 | m | no | no | no epilepsy |
| 38 | 2010 | f | no | no | no epilepsy |
| 39 | 2021 | m | no | no | no epilepsy |
| 40 | 2019 | m | no | no | no epilepsy |
| 41 | 2007 | m | no | no | no epilepsy |
| 42 | 2022 | f | no | no | no epilepsy |
| 43 | 2006 | m | no | no | no epilepsy |

**Suppl Table 5A: DP concentrations of the 43 individuals in CSF (fmol/µl)**

| patient | epilepsy | Glu-  Glu | Glu-  Ser | Car | Gly-  Glu | Val-  Tyr | Ala-  Ala | Asp-  Gln | Phe-  Ala | Ans | Gly-  Pro | Leu-  Pro | Ala-  Glu | Gly-  Asp | Pro-  Gly | γ-Glu-  ε-Lys | Gly-  Phe | Ser-  Ala | Ser-  Gln |
| --- | --- | --- | --- | --- | --- | --- | --- | --- | --- | --- | --- | --- | --- | --- | --- | --- | --- | --- | --- |
| 1 | focal | 0.22 | n.d. | n.d. | 0.59 | 0.10 | n.d. | 4.86 | n.d. | n.d. | 1.38 | n.d. | 1.05 | 10.22 | 0.50 | 6.76 | n.d. | n.d. | n.d. |
| 2 | focal | 2.04 | 3.76 | n.d. | 2.50 | 0.37 | 0.32 | n.d. | n.d. | 171.93 | 4.67 | n.d. | 0.14 | 52.69 | 1.12 | 11.22 | n.d. | n.d. | 2.49 |
| 3 | focal | n.d. | 0.68 | n.d. | 0.34 | n.d. | n.d. | 2.71 | n.d. | 29.77 | 2.36 | n.d. | 0.50 | 10.48 | 0.36 | n.d. | n.d. | n.d. | 0.74 |
| 4* | focal | n.d. | n.d. | n.d. | n.d. | n.d. | n.d. | n.d. | n.d. | 42.82 | 1.83 | n.d. | 0.98 | 16.47 | 0.66 | 22.87 | n.d. | n.d. | 1.43 |
| mean |  | ***1.13*** | ***2.22*** |  | ***1.14*** | ***0.23*** | ***0.32*** | ***3.79*** |  | ***81.50*** | ***2.56*** |  | ***0.67*** | ***22.46*** | ***0.66*** | ***13.62*** |  |  | ***1.55*** |
| SD |  | ***0.91*** | ***1.54*** |  | ***0.96*** | ***0.13*** | ***0.00*** | ***1.07*** |  | ***64.16*** | ***1.27*** |  | ***0.37*** | ***17.63*** | ***0.29*** | ***6.79*** |  |  | ***0.72*** |
| median |  | ***1.13*** | ***2.22*** |  | ***0.59*** | ***0.23*** | ***0.32*** | ***3.79*** |  | ***42.82*** | ***2.10*** |  | ***0.74*** | ***13.47*** | ***0.58*** | ***11.22*** |  |  | ***1.43*** |
|  |  |  |  |  |  |  |  |  |  |  |  |  |  |  |  |  |  |  |  |
| 5 | generalized | n.d. | 1.75 | n.d. | n.d. | 0.15 | 0.49 | n.d. | n.d. | 53.95 | 1.20 | n.d. | 0.77 | 19.54 | 0.81 | 2.72 | n.d. | n.d. | 0.98 |
| 6 | generalized | 0.20 | 1.34 | n.d. | 0.78 | 0.01 | 1.61 | 3.02 | n.d. | 69.72 | 5.44 | n.d. | 1.61 | 19.25 | 1.01 | n.d. | n.d. | n.d. | 1.88 |
| 7 | generalized | 0.63 | 2.22 | n.d. | 0.69 | 0.28 | n.d. | 5.76 | n.d. | n.d. | 5.21 | n.d. | 0.10 | 23.43 | 1.37 | 10.48 | n.d. | 0.34 | n.d. |
| 8 | generalized | n.d. | n.d. | n.d. | n.d. | 0.05 | n.d. | 7.03 | n.d. | n.d. | 3.87 | n.d. | 3.37 | 12.91 | 0.61 | 9.54 | n.d. | n.d. | 0.73 |
| 9 | generalized | n.d. | n.d. | n.d. | n.d. | n.d. | 0.54 | 5.97 | n.d. | 28.36 | 7.61 | 0.12 | 1.95 | 12.97 | 2.79 | 8.40 | 0.19 | 0.15 | 1.16 |
| 10 | generalized | 0.23 | n.d. | n.d. | 1.61 | 0.37 | 0.69 | 9.79 | n.d. | 128.31 | 6.48 | n.d. | 3.34 | 35.75 | 2.78 | 16.77 | n.d. | n.d. | 2.41 |
| 11 | generalized | n.d. | 1.46 | 11.35 | 0.94 | n.d. | 0.25 | 6.73 | n.d. | n.d. | 5.83 | n.d. | 1.10 | 17.85 | 0.86 | 12.64 | n.d. | n.d. | n.d. |
| 12* | generalized | n.d. | 1.39 | n.d. | 0.65 | n.d. | n.d. | 7.21 | n.d. | n.d. | 2.10 | n.d. | 0.90 | 12.14 | 0.90 | 20.65 | n.d. | n.d. | n.d. |
| 13 | generalized | 1.13 | 2.10 | n.d. | 1.59 | n.d. | 0.32 | 10.42 | n.d. | 95.29 | 11.03 | 0.05 | 7.45 | 32.95 | 2.17 | n.d. | n.d. | 0.40 | n.d. |
| 14 | generalized | 1.21 | 1.98 | n.d. | 1.27 | n.d. | 0.37 | n.d. | n.d. | 40.60 | 9.03 | 0.00 | 1.55 | 27.82 | 1.21 | 5.36 | n.d. | n.d. | 2.05 |
| mean |  | ***0.68*** | ***1.75*** | ***11.35*** | ***1.08*** | ***0.17*** | ***0.61*** | ***6.99*** |  | ***69.37*** | ***5.78*** | ***0.06*** | ***2.21*** | ***21.46*** | ***1.45*** | ***10.82*** | ***0.19*** | ***0.30*** | ***1.53*** |
| SD |  | ***0.43*** | ***0.33*** | ***0.00*** | ***0.38*** | ***0.13*** | ***0.43*** | ***2.18*** |  | ***33.90*** | ***2.84*** | ***0.05*** | ***2.01*** | ***7.96*** | ***0.78*** | ***5.46*** | ***0.00*** | ***0.10*** | ***0.61*** |
| median |  | ***0.63*** | ***1.75*** | ***11.35*** | ***0.94*** | ***0.15*** | ***0.49*** | ***6.88*** |  | ***61.83*** | ***5.63*** | ***0.05*** | ***1.58*** | ***19.39*** | ***1.11*** | ***10.01*** | ***0.19*** | ***0.34*** | ***1.52*** |
|  |  |  |  |  |  |  |  |  |  |  |  |  |  |  |  |  |  |  |  |
| 15 | unclear | 0.30 | n.d. | n.d. | 0.74 | n.d. | n.d. | 4.91 | n.d. | 48.15 | 4.30 | 0.04 | 2.01 | 16.25 | 1.20 | n.d. | n.d. | n.d. | n.d. |
| 16 | unclear | 0.15 | n.d. | n.d. | n.d. | 0.12 | n.d. | n.d. | n.d. | n.d. | 2.18 | n.d. | 1.61 | 10.89 | 0.59 | 16.56 | n.d. | n.d. | n.d. |
| 17 | unclear | n.d. | n.d. | n.d. | 0.81 | n.d. | n.d. | 7.34 | n.d. | n.d. | 2.52 | n.d. | 0.49 | 13.45 | 0.73 | 5.66 | n.d. | n.d. | n.d. |
| 18 | unclear | 0.84 | 9.43 | 29.81 | 3.80 | 0.51 | 3.74 | n.d. | 0.15 | 114.80 | 23.79 | 0.61 | 6.28 | 40.27 | 3.43 | 29.13 | 1.28 | 5.68 | 3.41 |
| 19 | unclear | 0.30 | n.d. | n.d. | 0.79 | 0.17 | 0.32 | 4.31 | n.d. | n.d. | 4.26 | 0.10 | 0.96 | 16.88 | 1.71 | 4.19 | n.d. | 0.49 | n.d. |
| 20 | unclear | n.d. | n.d. | n.d. | n.d. | 0.09 | 0.23 | n.d. | n.d. | n.d. | 2.57 | n.d. | 1.73 | 10.90 | 0.63 | 4.60 | n.d. | n.d. | 0.74 |
| 21 | unclear | n.d. | n.d. | n.d. | 0.76 | 0.09 | n.d. | n.d. | n.d. | n.d. | 4.39 | n.d. | 1.26 | 11.11 | n.d. | 9.46 | n.d. | n.d. | n.d. |
| 22 | unclear | n.d. | n.d. | n.d. | n.d. | n.d. | 0.65 | n.d. | n.d. | 26.56 | 3.49 | n.d. | 0.84 | 12.42 | 2.78 | 6.46 | n.d. | n.d. | 1.06 |
| 23* | unclear | n.d. | n.d. | n.d. | n.d. | n.d. | n.d. | 8.41 | n.d. | n.d. | 1.63 | n.d. | 1.42 | 9.16 | 0.91 | 13.81 | n.d. | n.d. | n.d. |
| mean |  | ***0.40*** | ***9.43*** | ***29.81*** | ***1.38*** | ***0.20*** | ***1.23*** | ***6.24*** | ***0.15*** | ***63.17*** | ***5.46*** | ***0.25*** | ***1.85*** | ***15.70*** | ***1.50*** | ***11.23*** | ***1.28*** | ***3.09*** | ***1.74*** |
| SD |  | ***0.26*** | ***0.00*** | ***0.00*** | ***1.21*** | ***0.16*** | ***1.46*** | ***1.69*** | ***0.00*** | ***37.56*** | ***6.55*** | ***0.26*** | ***1.63*** | ***9.01*** | ***1.00*** | ***7.95*** | ***0.00*** | ***2.59*** | ***1.19*** |
| median |  | ***0.30*** | ***9.43*** | ***29.81*** | ***0.79*** | ***0.12*** | ***0.49*** | ***6.12*** | ***0.15*** | ***48.15*** | ***3.49*** | ***0.10*** | ***1.42*** | ***12.42*** | ***1.06*** | ***7.96*** | ***1.28*** | ***3.09*** | ***1.06*** |
|  |  |  |  |  |  |  |  |  |  |  |  |  |  |  |  |  |  |  |  |
| 24 | no epilepsy | 0.09 | 1.25 | n.d. | 0.77 | n.d. | 0.78 | 5.07 | n.d. | 39.76 | 3.96 | n.d. | 1.14 | 18.09 | 0.54 | 9.89 | n.d. | 0.57 | n.d. |
| 25 | no epilepsy | n.d. | n.d. | n.d. | n.d. | n.d. | 0.17 | 6.66 | n.d. | 22.88 | 1.43 | n.d. | 0.70 | 19.17 | 1.06 | 15.00 | n.d. | n.d. | n.d. |
| 26 | no epilepsy | 0.35 | n.d. | n.d. | 0.88 | n.d. | 0.18 | 7.63 | n.d. | 58.61 | 3.56 | n.d. | 1.58 | 18.55 | 1.04 | 5.90 | n.d. | 0.23 | n.d. |
| 27 | no epilepsy | n.d. | n.d. | n.d. | n.d. | n.d. | 0.74 | n.d. | n.d. | n.d. | 1.95 | n.d. | 3.17 | 10.09 | 0.86 | 22.11 | n.d. | n.d. | n.d. |
| 28 | no epilepsy | n.d. | 1.50 | n.d. | n.d. | 0.12 | 0.48 | 11.92 | 0.02 | n.d. | 6.93 | 0.14 | 0.38 | 23.05 | 2.02 | 8.09 | n.d. | n.d. | n.d. |
| 29 | no epilepsy | n.d. | n.d. | n.d. | n.d. | n.d. | n.d. | n.d. | n.d. | 22.60 | 4.30 | 0.06 | 1.10 | 11.52 | 1.43 | 14.92 | n.d. | n.d. | n.d. |
| 30 | no epilepsy | 0.38 | n.d. | n.d. | n.d. | n.d. | 1.67 | 7.81 | n.d. | n.d. | 2.74 | n.d. | 1.15 | 16.11 | 1.34 | 20.86 | n.d. | n.d. | n.d. |
| 31 | no epilepsy | n.d. | 1.36 | 10.81 | 2.09 | 0.08 | 0.14 | n.d. | n.d. | 91.70 | 8.76 | 0.01 | 2.24 | 26.09 | 1.53 | 22.19 | n.d. | 0.21 | n.d. |
| 32 | no epilepsy | 0.72 | 1.34 | n.d. | n.d. | 0.03 | n.d. | 5.03 | n.d. | 24.09 | 1.40 | n.d. | 0.72 | 10.83 | 1.02 | 9.48 | n.d. | n.d. | 1.16 |
| 33 | no epilepsy | 0.92 | 1.71 | n.d. | n.d. | 0.11 | 1.35 | n.d. | n.d. | n.d. | 4.22 | n.d. | 1.79 | 18.18 | 1.79 | 16.11 | n.d. | 0.45 | n.d. |
| 34 | no epilepsy | n.d. | 1.04 | n.d. | 0.70 | 0.05 | 0.46 | 3.78 | n.d. | 32.64 | 2.39 | n.d. | 1.16 | 13.29 | 2.09 | 15.64 | n.d. | 0.20 | n.d. |
| 35 | no epilepsy | 1.17 | n.d. | n.d. | 1.17 | n.d. | n.d. | 5.40 | n.d. | n.d. | 3.01 | 0.06 | 0.90 | 14.30 | 0.87 | 20.60 | n.d. | n.d. | 2.01 |
| 36 | no epilepsy | 0.25 | n.d. | 4.43 | n.d. | 0.06 | n.d. | 6.46 | n.d. | 53.98 | 3.53 | n.d. | 1.53 | 13.75 | 2.83 | 1.25 | n.d. | 0.42 | 1.72 |
| 37 | no epilepsy | 3.36 | 3.78 | n.d. | 0.89 | 0.25 | 1.42 | n.d. | 0.04 | 70.60 | 3.74 | 0.67 | 2.24 | 19.43 | 1.54 | n.d. | 0.08 | n.d. | 1.90 |
| 38 | no epilepsy | n.d. | 1.30 | n.d. | 0.43 | 0.03 | 0.22 | n.d. | n.d. | 17.73 | 3.96 | 0.01 | 1.11 | 16.95 | 0.95 | 0.19 | n.d. | n.d. | 1.95 |
| 39 | no epilepsy | n.d. | 2.45 | n.d. | n.d. | n.d. | n.d. | 4.33 | n.d. | n.d. | 2.80 | 0.02 | 0.53 | 12.31 | 0.94 | n.d. | n.d. | 0.44 | n.d. |
| 40 | no epilepsy | 0.23 | n.d. | n.d. | n.d. | n.d. | 5.12 | n.d. | n.d. | n.d. | 3.41 | n.d. | 0.79 | 14.16 | 0.95 | 19.07 | n.d. | n.d. | n.d. |
| 41 | no epilepsy | n.d. | n.d. | n.d. | n.d. | n.d. | n.d. | 5.88 | n.d. | 25.76 | 3.11 | n.d. | 0.88 | 13.22 | 0.61 | n.d. | n.d. | n.d. | n.d. |
| 42 | no epilepsy | n.d. | 1.62 | n.d. | 1.42 | 0.28 | n.d. | 6.26 | n.d. | n.d. | 6.50 | n.d. | 2.02 | 16.29 | 1.47 | 21.85 | n.d. | 0.28 | n.d. |
| 43 | no epilepsy | 0.10 | 2.20 | n.d. | n.d. | 0.04 | 0.23 | n.d. | n.d. | 24.40 | 3.89 | n.d. | 0.16 | 16.90 | 0.71 | 11.66 | n.d. | n.d. | n.d. |
| mean |  | ***0.76*** | ***1.78*** | ***7.62*** | ***1.05*** | ***0.11*** | ***1.00*** | ***6.35*** | ***0.03*** | ***40.40*** | ***3.78*** | ***0.14*** | ***1.27*** | ***16.11*** | ***1.28*** | ***13.81*** | ***0.08*** | ***0.35*** | ***1.75*** |
| SD |  | ***0.93*** | ***0.75*** | ***3.19*** | ***0.48*** | ***0.09*** | ***1.29*** | ***2.04*** | ***0.01*** | ***22.35*** | ***1.77*** | ***0.22*** | ***0.72*** | ***3.95*** | ***0.56*** | ***6.93*** | ***0.00*** | ***0.13*** | ***0.31*** |
| median |  | ***0.36*** | ***1.50*** | ***7.62*** | ***0.89*** | ***0.07*** | ***0.48*** | ***6.07*** | ***0.03*** | ***29.20*** | ***3.54*** | ***0.06*** | ***1.13*** | ***16.20*** | ***1.05*** | ***15.00*** | ***0.08*** | ***0.35*** | ***1.90*** |

Mean with standard deviation (SD) and median was calculated for the different clinical groups (focal epilepsy, generalized epilepsy, unclear epilepsy, no epilepsy).
* = patients that received valproate
n.d. = compounds that could not be determined due to low detection (below baseline), a very poor signal-to-noise ratio or strong overlap with an unknown co-eluting peak

**Suppl. Table 5B: DP concentrations of the 43 individuals in plasma (fmol/µl)**

| patient | epilepsy | Glu-  Glu | Glu-  Ser | Car | Gly-  Glu | Val-  Tyr | Ala-  Ala | Asp-  Gln | Phe-  Ala | Ans | Gly-  Pro | Ala-  Glu | Gly-  Asp | Pro-  Gly | γ-Glu-  ε-Lys | Gly-  Phe | Ser-  Ala | Ser-  Gln | Ala-  Gly | Ala-  Phe | Ala-  Pro |
| --- | --- | --- | --- | --- | --- | --- | --- | --- | --- | --- | --- | --- | --- | --- | --- | --- | --- | --- | --- | --- | --- |
| 1 | focal | n.d. | 3.12 | n.d. | 5.11 | 1.11 | 1.39 | n.d. | 0.46 | n.d. | 2.99 | 5.53 | 21.45 | 6.51 | n.d. | 0.78 | n.d. | n.d. | n.d. | n.d. | 0.65 |
| 2 | focal | 0.52 | 2.46 | 357.03 | 16.41 | 1.10 | 4.03 | 25.35 | 0.31 | n.d. | 6.08 | 3.21 | 149.68 | n.d. | n.d. | n.d. | n.d. | n.d. | n.d. | n.d. | 2.62 |
| 3 | focal | 3.69 | n.d. | 46.14 | 7.55 | n.d. | 2.79 | 6.14 | n.d. | n.d. | 0.58 | 9.41 | 30.09 | n.d. | n.d. | n.d. | n.d. | n.d. | 5.13 | n.d. | n.d. |
| 4* | focal | 21.39 | n.d. | n.d. | 5.11 | n.d. | 1.07 | n.d. | n.d. | n.d. | 4.81 | 19.86 | 43.19 | 4.39 | n.d. | n.d. | 0.63 | 3.94 | 1.54 | n.d. | 0.34 |
| mean |  | ***8.54*** | ***2.79*** | ***201.58*** | ***8.54*** | ***1.11*** | ***2.32*** | ***15.74*** | ***0.39*** |  | ***3.61*** | ***9.50*** | ***61.10*** | ***5.45*** | ***n.d.*** | ***0.78*** | ***0.63*** | ***3.94*** | ***3.34*** |  | ***1.21*** |
| SD |  | ***9.18*** | ***0.33*** | ***155.44*** | ***4.65*** | ***0.00*** | ***1.18*** | ***9.61*** | ***0.07*** |  | ***2.07*** | ***6.38*** | ***51.72*** | ***1.06*** | ***n.d.*** | ***0.00*** | ***0.00*** | ***0.00*** | ***1.79*** |  | ***1.01*** |
| median |  | ***3.69*** | ***2.79*** | ***201.58*** | ***6.33*** | ***1.11*** | ***2.09*** | ***15.74*** | ***0.39*** |  | ***3.90*** | ***7.47*** | ***36.64*** | ***5.45*** | ***n.d.*** | ***0.78*** | ***0.63*** | ***3.94*** | ***3.34*** |  | ***0.65*** |
|  |  |  |  |  |  |  |  |  |  |  |  |  |  |  |  |  |  |  |  |  |  |
| 5 | generalized | 25.25 | 5.60 | 194.74 | 17.03 | 0.52 | n.d. | n.d. | n.d. | 77.33 | 6.31 | 23.23 | 73.75 | n.d. | n.d. | n.d. | 4.89 | n.d. | 1.01 | n.d. | 0.26 |
| 6 | generalized | 30.88 | 7.39 | 580.03 | 21.91 | 0.33 | 20.61 | 23.45 | 1.45 | 68.28 | 11.85 | 30.62 | 61.51 | 6.35 | n.d. | 0.83 | 2.48 | n.d. | 7.22 | n.d. | 1.09 |
| 7 | generalized | 8.97 | n.d. | n.d. | 8.75 | n.d. | n.d. | n.d. | 0.25 | n.d. | 3.00 | 8.32 | 37.64 | 3.96 | n.d. | n.d. | 2.30 | 3.24 | 4.87 | n.d. | n.d. |
| 8 | generalized | 3.20 | n.d. | 79.38 | 4.82 | n.d. | 1.39 | n.d. | n.d. | n.d. | 0.51 | 9.26 | 22.97 | 1.24 | n.d. | n.d. | 2.19 | n.d. | 1.43 | n.d. | n.d. |
| 9 | generalized | 8.65 | 2.96 | n.d. | 5.39 | 0.00 | 0.87 | n.d. | n.d. | 103.65 | 2.30 | 13.28 | 46.42 | 1.75 | n.d. | n.d. | n.d. | n.d. | n.d. | n.d. | n.d. |
| 10 | generalized | 34.16 | 5.92 | 314.40 | 16.51 | n.d. | 4.95 | n.d. | n.d. | n.d. | 6.02 | 31.45 | 70.62 | 21.48 | n.d. | 0.93 | 2.17 | n.d. | 25.50 | n.d. | n.d. |
| 11 | generalized | 4.94 | n.d. | 22.73 | 9.32 | 0.27 | 2.07 | n.d. | n.d. | n.d. | 5.05 | 11.91 | 25.90 | 2.77 | n.d. | n.d. | 1.82 | n.d. | 2.85 | n.d. | 1.01 |
| 12* | generalized | 14.44 | n.d. | n.d. | 4.40 | n.d. | n.d. | n.d. | n.d. | n.d. | 0.94 | 16.25 | 40.80 | 3.23 | n.d. | n.d. | n.d. | 2.76 | 1.17 | n.d. | 0.44 |
| 13 | generalized | 44.96 | 27.38 | n.d. | 18.13 | n.d. | 1.11 | 160.91 | n.d. | n.d. | 17.96 | 66.73 | 65.60 | 8.53 | 27.26 | 33.53 | 139.15 | 8.91 | 12.71 | n.d. | 0.36 |
| 14 | generalized | 12.70 | n.d. | n.d. | 3.49 | n.d. | n.d. | n.d. | n.d. | n.d. | 3.28 | 37.97 | 41.24 | 4.25 | n.d. | n.d. | n.d. | n.d. | 2.53 | n.d. | 1.06 |
| mean |  | ***18.82*** | ***9.85*** | ***238.25*** | ***10.97*** | ***0.28*** | ***5.17*** | ***92.18*** | ***0.85*** | ***83.09*** | ***5.72*** | ***24.90*** | ***48.64*** | ***5.95*** | ***27.26*** | ***11.77*** | ***22.14*** | ***4.97*** | ***6.59*** |  | ***0.71*** |
| SD |  | ***13.41*** | ***8.88*** | ***198.09*** | ***6.43*** | ***0.18*** | ***7.04*** | ***68.73*** | ***0.60*** | ***15.01*** | ***5.13*** | ***17.02*** | ***17.28*** | ***5.89*** | ***0.00*** | ***15.39*** | ***47.78*** | ***2.79*** | ***7.57*** |  | ***0.35*** |
| median |  | ***13.57*** | ***5.92*** | ***194.74*** | ***9.04*** | ***0.30*** | ***1.73*** | ***92.18*** | ***0.85*** | ***77.33*** | ***4.16*** | ***19.74*** | ***43.83*** | ***3.96*** | ***27.26*** | ***0.93*** | ***2.30*** | ***3.24*** | ***2.85*** |  | ***0.73*** |
|  |  |  |  |  |  |  |  |  |  |  |  |  |  |  |  |  |  |  |  |  |  |
| 15 | unclear | n.d. | 5.68 | 159.28 | 17.68 | 0.42 | 3.10 | 15.72 | n.d. | n.d. | 16.08 | 3.34 | 70.99 | 7.50 | n.d. | 0.57 | 4.35 | n.d. | 10.43 | 0.17 | 0.53 |
| 16 | unclear | n.d. | 2.16 | n.d. | 12.30 | 0.32 | 1.20 | n.d. | 0.79 | n.d. | 1.20 | 10.31 | 56.17 | 4.12 | n.d. | n.d. | 1.42 | n.d. | 4.14 | n.d. | 0.56 |
| 17 | unclear | 20.86 | 3.70 | 46.12 | 7.87 | 0.31 | 2.72 | n.d. | 1.12 | n.d. | 6.30 | 14.44 | 73.24 | 4.47 | n.d. | n.d. | 4.32 | 11.04 | n.d. | n.d. | n.d. |
| 18 | unclear | 43.21 | 3.98 | 1858.54 | 30.04 | 0.93 | 7.51 | 16.26 | 0.98 | 127.88 | 32.28 | 47.60 | 141.21 | 12.19 | n.d. | 0.23 | n.d. | 3.56 | 10.54 | n.d. | 2.52 |
| 19 | unclear | n.d. | n.d. | n.d. | 6.31 | n.d. | 1.91 | n.d. | 2.26 | n.d. | 2.78 | 12.29 | 36.78 | 4.34 | n.d. | 0.88 | 4.57 | 3.03 | 5.04 | n.d. | n.d. |
| 20 | unclear | 23.62 | 3.07 | n.d. | 5.24 | n.d. | n.d. | 22.92 | n.d. | n.d. | 1.10 | 1.68 | 40.03 | 2.01 | n.d. | n.d. | 1.65 | n.d. | n.d. | n.d. | 0.68 |
| 21 | unclear | 6.00 | 3.31 | n.d. | 14.06 | 0.19 | 0.83 | 16.81 | n.d. | n.d. | 1.61 | 28.72 | 56.48 | 3.20 | n.d. | n.d. | 2.69 | n.d. | 0.94 | n.d. | 0.59 |
| 22 | unclear | 20.15 | n.d. | n.d. | n.d. | 0.16 | 3.40 | n.d. | n.d. | 68.82 | 0.64 | 10.57 | 52.29 | 2.30 | n.d. | n.d. | 1.23 | n.d. | n.d. | n.d. | 0.37 |
| 23* | unclear | 46.17 | 2.11 | n.d. | n.d. | n.d. | n.d. | n.d. | n.d. | n.d. | 2.67 | 48.74 | 37.11 | 2.81 | n.d. | n.d. | n.d. | n.d. | 9.36 | n.d. | n.d. |
| mean |  | ***26.67*** | ***3.43*** | ***687.98*** | ***13.36*** | ***0.39*** | ***2.95*** | ***17.93*** | ***1.29*** | ***98.35*** | ***7.18*** | ***19.74*** | ***62.70*** | ***4.77*** | ***n.d.*** | ***0.56*** | ***2.89*** | ***5.88*** | ***6.74*** | ***0.17*** | ***0.88*** |
| SD |  | ***13.94*** | ***1.13*** | ***829.00*** | ***7.97*** | ***0.26*** | ***2.06*** | ***2.91*** | ***0.57*** | ***29.53*** | ***9.98*** | ***16.83*** | ***30.50*** | ***3.04*** | ***n.d.*** | ***0.27*** | ***1.39*** | ***3.66*** | ***3.61*** | ***0.00*** | ***0.74*** |
| median |  | ***22.24*** | ***3.31*** | ***159.28*** | ***12.30*** | ***0.31*** | ***2.72*** | ***16.54*** | ***1.05*** | ***98.35*** | ***2.67*** | ***12.29*** | ***56.17*** | ***4.12*** | ***n.d.*** | ***0.57*** | ***2.69*** | ***3.56*** | ***7.20*** | ***0.17*** | ***0.58*** |
|  |  |  |  |  |  |  |  |  |  |  |  |  |  |  |  |  |  |  |  |  |  |
| 24 | no epilepsy | n.d. | 2.35 | n.d. | n.d. | 0.16 | n.d. | n.d. | 1.44 | n.d. | 3.79 | 2.47 | 32.33 | 4.36 | 35.45 | n.d. | n.d. | 4.60 | 4.41 | n.d. | 0.59 |
| 25 | no epilepsy | n.d. | n.d. | n.d. | n.d. | 0.40 | 0.81 | n.d. | 0.02 | 154.47 | 0.61 | 7.52 | 54.29 | 3.73 | n.d. | n.d. | n.d. | n.d. | n.d. | n.d. | 2.20 |
| 26 | no epilepsy | 7.29 | n.d. | n.d. | 7.98 | 0.36 | n.d. | n.d. | n.d. | n.d. | 2.84 | 13.51 | 38.82 | 3.74 | 13.13 | n.d. | 1.04 | n.d. | 3.29 | n.d. | n.d. |
| 27 | no epilepsy | 10.61 | n.d. | n.d. | n.d. | n.d. | 3.37 | n.d. | n.d. | n.d. | 1.12 | 40.60 | 24.53 | 3.82 | n.d. | n.d. | n.d. | 1.96 | n.d. | n.d. | n.d. |
| 28 | no epilepsy | 18.32 | n.d. | n.d. | 2.17 | n.d. | 2.49 | n.d. | n.d. | n.d. | 1.58 | 27.71 | 30.03 | 4.01 | n.d. | n.d. | n.d. | 3.14 | n.d. | n.d. | n.d. |
| 29 | no epilepsy | 3.37 | n.d. | n.d. | 15.87 | 0.36 | 2.06 | 19.77 | 3.21 | 82.00 | 28.02 | 33.05 | 39.08 | 6.26 | n.d. | n.d. | 1.77 | n.d. | 14.93 | n.d. | 0.12 |
| 30 | no epilepsy | 2.42 | 2.46 | n.d. | 3.98 | 0.00 | 7.99 | 10.11 | 0.54 | 43.27 | 0.86 | 14.92 | 25.57 | 2.69 | n.d. | n.d. | n.d. | n.d. | 3.14 | n.d. | n.d. |
| 31 | no epilepsy | 2.17 | 3.73 | 116.27 | 19.03 | 0.15 | n.d. | n.d. | 0.55 | 82.22 | 12.35 | n.d. | 73.81 | 5.77 | n.d. | n.d. | n.d. | n.d. | 7.74 | n.d. | 0.88 |
| 32 | no epilepsy | 10.08 | 2.14 | n.d. | 7.88 | n.d. | 4.04 | n.d. | 0.73 | n.d. | 1.17 | 13.45 | 38.93 | 3.47 | n.d. | n.d. | 1.82 | n.d. | n.d. | n.d. | n.d. |
| 33 | no epilepsy | 3.78 | n.d. | n.d. | 2.10 | 0.12 | 2.55 | n.d. | n.d. | n.d. | 0.49 | 22.87 | 21.74 | 5.17 | n.d. | n.d. | n.d. | n.d. | n.d. | n.d. | n.d. |
| 34 | no epilepsy | 17.86 | 2.15 | n.d. | 7.04 | n.d. | 1.53 | n.d. | n.d. | 111.20 | 1.03 | 15.76 | 51.34 | 2.61 | n.d. | n.d. | n.d. | n.d. | 2.66 | n.d. | n.d. |
| 35 | no epilepsy | 2.98 | 1.86 | n.d. | 3.45 | n.d. | n.d. | n.d. | 0.43 | n.d. | 2.54 | 10.78 | 19.22 | 3.78 | n.d. | n.d. | 1.47 | n.d. | n.d. | n.d. | n.d. |
| 36 | no epilepsy | 18.72 | 29.73 | n.d. | 6.75 | n.d. | n.d. | 70.73 | n.d. | 37.28 | 3.24 | 20.16 | 41.72 | n.d. | n.d. | 14.14 | 228.20 | 21.24 | n.d. | n.d. | n.d. |
| 37 | no epilepsy | 25.41 | 70.84 | n.d. | 6.97 | n.d. | n.d. | 145.99 | n.d. | n.d. | 2.96 | 46.35 | 70.54 | 6.48 | n.d. | 76.63 | 73.44 | n.d. | n.d. | n.d. | 0.55 |
| 38 | no epilepsy | 1.12 | n.d. | n.d. | 4.44 | 0.03 | n.d. | 21.88 | n.d. | n.d. | 3.43 | 14.40 | 32.45 | 2.98 | n.d. | n.d. | n.d. | n.d. | 1.20 | n.d. | 0.12 |
| 39 | no epilepsy | 48.00 | n.d. | n.d. | 4.32 | n.d. | n.d. | n.d. | n.d. | n.d. | n.d. | 26.60 | 19.60 | 2.97 | n.d. | n.d. | 2.73 | n.d. | n.d. | n.d. | n.d. |
| 40 | no epilepsy | 8.73 | 3.67 | n.d. | 6.35 | n.d. | 19.29 | n.d. | n.d. | n.d. | 1.62 | 14.96 | 48.36 | 4.05 | n.d. | n.d. | n.d. | n.d. | 1.55 | n.d. | 0.61 |
| 41 | no epilepsy | 14.60 | n.d. | n.d. | 4.01 | n.d. | n.d. | 11.11 | n.d. | 73.57 | 8.39 | 44.94 | 43.49 | 5.56 | n.d. | n.d. | n.d. | n.d. | 1.42 | n.d. | n.d. |
| 42 | no epilepsy | 23.07 | 4.63 | 125.96 | 11.52 | n.d. | 0.32 | n.d. | n.d. | n.d. | 11.11 | 37.06 | 61.61 | 2.91 | n.d. | n.d. | n.d. | n.d. | n.d. | n.d. | 0.47 |
| 43 | no epilepsy | 10.56 | 2.52 | n.d. | n.d. | 0.67 | 1.97 | n.d. | n.d. | n.d. | 13.99 | 29.75 | 87.06 | 20.66 | n.d. | n.d. | 1.34 | n.d. | 12.10 | n.d. | 4.20 |
| mean |  | ***12.73*** | ***11.46*** | ***121.12*** | ***7.11*** | ***0.25*** | ***4.22*** | ***46.60*** | ***0.99*** | ***83.43*** | ***5.32*** | ***22.99*** | ***42.73*** | ***5.00*** | ***24.29*** | ***45.38*** | ***38.98*** | ***7.74*** | ***5.24*** |  | ***1.08*** |
| SD |  | ***11.27*** | ***20.31*** | ***4.85*** | ***4.59*** | ***0.20*** | ***5.15*** | ***48.96*** | ***0.99*** | ***37.15*** | ***6.72*** | ***12.51*** | ***18.54*** | ***3.87*** | ***11.16*** | ***31.24*** | ***75.28*** | ***7.86*** | ***4.55*** |  | ***1.25*** |
| median |  | ***10.32*** | ***2.52*** | ***121.12*** | ***6.55*** | ***0.16*** | ***2.49*** | ***20.82*** | ***0.55*** | ***82.00*** | ***2.84*** | ***20.16*** | ***39.00*** | ***3.82*** | ***24.29*** | ***45.38*** | ***1.80*** | ***3.87*** | ***3.21*** |  | ***0.59*** |

Mean with standard deviation (SD) and median was calculated for the different clinical groups (focal epilepsy, generalized epilepsy, unclear epilepsy, no epilepsy).
* = patients that received valproate
n.d. = compounds that could not be determined due to low detection (below baseline), a very poor signal-to-noise ratio or strong overlap with an unknown co-eluting peak.

**Suppl. Table 5C: AA concentrations of the 43 individuals in CSF (pmol/µl)**

| patient | epilepsy | Ala | Arg | Asn | Gln | Asp | Glu | His | Tyr | Val | Gly | Ile | Met | Phe | Pro | Leu | Lys | Ser | Thr |  |
| --- | --- | --- | --- | --- | --- | --- | --- | --- | --- | --- | --- | --- | --- | --- | --- | --- | --- | --- | --- | --- |
| 1 | focal | 16.87 | 18.10 | 3.64 | 236.10 | n.d. | n.d. | 8.23 | 8.52 | 10.72 | 18.54 | 2.99 | 1.09 | 5.24 | n.d. | 7.99 | 16.05 | 32.39 | 18.59 | |
| 2 | focal | 35.96 | 12.81 | 14.70 | 415.14 | n.d. | 1.19 | 35.06 | 31.06 | 51.25 | 21.82 | 17.30 | 11.44 | 125.05 | 5.56 | 40.27 | 26.85 | 37.31 | 39.98 | |
| 3 | focal | 10.88 | 10.01 | 3.39 | 215.87 | n.d. | 0.02 | 7.36 | 5.01 | 12.65 | 18.51 | 3.79 | 0.51 | 6.57 | n.d. | 9.00 | 11.75 | 18.87 | 7.59 | |
| 4* | focal | 21.37 | 14.49 | 4.95 | 309.66 | n.d. | 0.38 | 12.58 | 8.85 | 18.02 | 20.92 | 3.42 | 1.85 | 7.48 | 0.11 | 10.63 | 19.19 | 28.92 | 19.30 | |
| mean |  | ***21.27*** | ***13.85*** | ***6.67*** | ***294.19*** |  | ***0.53*** | ***15.81*** | ***13.36*** | ***23.16*** | ***19.95*** | ***6.87*** | ***3.72*** | ***36.09*** | ***2.83*** | ***16.97*** | ***18.46*** | ***29.37*** | ***21.36*** | |
| SD |  | ***9.26*** | ***2.93*** | ***4.67*** | ***78.06*** |  | ***0.49*** | ***11.29*** | ***10.33*** | ***16.44*** | ***1.46*** | ***6.03*** | ***4.48*** | ***51.37*** | ***2.72*** | ***13.48*** | ***5.52*** | ***6.76*** | ***11.71*** | |
| median |  | ***19.12*** | ***13.65*** | ***4.29*** | ***272.88*** |  | ***0.38*** | ***10.41*** | ***8.69*** | ***15.34*** | ***19.73*** | ***3.60*** | ***1.47*** | ***7.03*** | ***2.83*** | ***9.81*** | ***17.62*** | ***30.66*** | ***18.94*** | |
|  |  |  |  |  |  |  |  |  |  |  |  |  |  |  |  |  |  |  |  | |
| 5 | generalized | 22.68 | 12.92 | 5.11 | 203.90 | n.d. | 0.73 | 7.67 | 7.36 | 11.78 | 16.31 | 3.99 | 0.73 | 5.79 | 0.22 | 9.82 | 16.42 | 29.14 | 15.84 | |
| 6 | generalized | 24.14 | 17.23 | 7.81 | 288.21 | n.d. | 0.48 | 12.97 | 19.36 | 16.70 | 17.78 | 6.62 | 3.07 | 9.02 | 0.84 | 15.93 | 19.55 | 43.69 | 44.11 | |
| 7 | generalized | 20.32 | 14.56 | 5.94 | 246.41 | n.d. | 0.37 | 10.36 | 4.91 | 17.41 | 18.73 | 5.07 | 1.82 | 6.42 | 0.10 | 13.41 | 23.45 | 21.71 | 28.52 | |
| 8 | generalized | 29.76 | 18.20 | 6.39 | 279.27 | n.d. | 1.02 | 8.98 | 6.78 | 15.00 | 18.12 | 5.28 | 1.78 | 8.46 | 1.61 | 13.26 | 22.74 | 39.67 | 30.21 | |
| 9 | generalized | 25.29 | 16.22 | 7.33 | 244.28 | 1.01 | 1.07 | 8.62 | 10.94 | 9.47 | 35.86 | 3.11 | 1.86 | 6.10 | 4.35 | 6.58 | 15.02 | 45.69 | 27.58 | |
| 10 | generalized | 39.12 | 27.15 | 13.36 | 334.54 | 0.52 | 1.11 | 21.40 | 12.81 | 25.93 | 23.46 | 10.45 | 2.72 | 10.67 | 1.66 | 23.84 | 38.31 | 54.42 | 48.48 | |
| 11 | generalized | 24.97 | 13.43 | 6.26 | 268.58 | 0.67 | 1.36 | 9.05 | 9.28 | 21.74 | 23.77 | 6.62 | 2.13 | 7.63 | 1.53 | 16.46 | 23.75 | 49.97 | 25.56 | |
| 12* | generalized | 14.28 | 10.14 | 3.67 | 237.67 | n.d. | n.d. | 7.39 | 7.73 | 10.38 | 15.37 | 2.48 | 1.05 | 5.81 | n.d. | 7.29 | 12.42 | 23.39 | 13.75 | |
| 13 | generalized | 55.04 | 43.53 | 12.26 | 347.61 | n.d. | 1.23 | 14.64 | 19.32 | 24.34 | 26.46 | 7.63 | 7.07 | 16.24 | 1.97 | 17.43 | 33.42 | 63.90 | 58.09 | |
| 14 | generalized | 42.82 | 30.37 | 12.40 | 337.90 | 0.17 | 3.56 | 17.36 | 14.77 | 22.18 | 49.27 | 6.57 | 4.19 | 12.43 | 8.23 | 13.66 | 39.52 | 50.61 | 56.50 | |
| mean |  | ***29.84*** | ***20.38*** | ***8.05*** | ***278.84*** | ***0.59*** | ***1.21*** | ***11.84*** | ***11.33*** | ***17.49*** | ***24.51*** | ***5.78*** | ***2.64*** | ***8.86*** | ***2.28*** | ***13.77*** | ***24.46*** | ***42.22*** | ***34.86*** | |
| SD |  | ***11.62*** | ***9.78*** | ***3.22*** | ***45.90*** | ***0.30*** | ***0.89*** | ***4.43*** | ***4.87*** | ***5.59*** | ***10.09*** | ***2.22*** | ***1.75*** | ***3.23*** | ***2.41*** | ***4.86*** | ***9.09*** | ***13.10*** | ***15.12*** | |
| median |  | ***25.13*** | ***16.73*** | ***6.86*** | ***273.93*** | ***0.59*** | ***1.07*** | ***9.71*** | ***10.11*** | ***17.05*** | ***21.09*** | ***5.92*** | ***1.99*** | ***8.05*** | ***1.61*** | ***13.54*** | ***23.10*** | ***44.69*** | ***29.36*** | |
|  |  |  |  |  |  |  |  |  |  |  |  |  |  |  |  |  |  |  |  | |
| 15 | unclear | 28.92 | 23.63 | 7.52 | 280.57 | n.d. | 0.12 | 13.38 | 13.58 | 11.83 | 17.51 | 2.91 | 3.46 | 10.62 | 0.78 | 7.20 | 24.83 | 45.93 | 33.28 | |
| 16 | unclear | 20.55 | 14.20 | 4.84 | 235.51 | n.d. | n.d. | 10.51 | 8.81 | 12.09 | 15.42 | 3.30 | 1.45 | 7.82 | 0.18 | 8.90 | 15.08 | 29.95 | 21.13 | |
| 17 | unclear | n.d. | 10.85 | 5.20 | 236.66 | n.d. | 0.10 | 8.41 | 4.29 | 12.58 | n.d. | 4.24 | 1.01 | 4.91 | 0.22 | 10.93 | 10.02 | n.d. | 15.99 | |
| 18 | unclear | 54.47 | 24.48 | 18.75 | 327.78 | 14.12 | 6.09 | 30.82 | 28.42 | 41.01 | 54.67 | 16.54 | 4.23 | 14.88 | 9.18 | 32.15 | 39.18 | 104.16 | 65.67 | |
| 19 | unclear | 18.04 | 12.49 | 5.61 | 260.46 | 1.43 | 0.92 | 11.48 | 10.54 | 10.15 | 21.07 | 3.44 | 2.54 | 6.94 | 0.73 | 8.39 | 15.87 | 30.78 | 17.64 | |
| 20 | unclear | 29.09 | 21.33 | 7.10 | 287.38 | n.d. | 0.18 | 12.31 | 7.25 | 16.96 | 20.95 | 4.43 | 1.98 | 7.52 | n.d. | 10.57 | 19.76 | 34.73 | 25.09 | |
| 21 | unclear | 21.04 | 14.46 | 3.65 | 256.29 | n.d. | 0.25 | 10.42 | 5.33 | 18.29 | 17.62 | 5.08 | 3.44 | 10.34 | n.d. | 12.89 | 21.70 | 23.44 | 35.92 | |
| 22 | unclear | 14.38 | 11.33 | 4.20 | 189.50 | 0.05 | 0.06 | 5.74 | 7.81 | 11.15 | 18.77 | 2.08 | 0.75 | 4.39 | 0.11 | 5.69 | 14.18 | 23.29 | 16.82 | |
| 23* | unclear | 23.33 | 12.58 | 6.47 | 312.14 | n.d. | 0.55 | 13.42 | 10.04 | 8.97 | 17.69 | 3.15 | 2.97 | 7.24 | n.d. | 8.16 | 14.50 | 23.84 | 27.27 | |
| mean |  | ***26.23*** | ***16.15*** | ***7.04*** | ***265.14*** | ***5.20*** | ***1.03*** | ***12.94*** | ***10.67*** | ***15.89*** | ***22.96*** | ***5.02*** | ***2.43*** | ***8.30*** | ***1.87*** | ***11.65*** | ***19.46*** | ***39.52*** | ***28.76*** | |
| SD |  | ***11.66*** | ***5.12*** | ***4.31*** | ***39.97*** | ***6.33*** | ***1.93*** | ***6.73*** | ***6.80*** | ***9.33*** | ***12.11*** | ***4.16*** | ***1.14*** | ***3.05*** | ***3.28*** | ***7.52*** | ***8.14*** | ***25.44*** | ***14.67*** | |
| median |  | ***22.18*** | ***14.20*** | ***5.61*** | ***260.46*** | ***1.43*** | ***0.21*** | ***11.48*** | ***8.81*** | ***12.09*** | ***18.23*** | ***3.44*** | ***2.54*** | ***7.52*** | ***0.48*** | ***8.90*** | ***15.87*** | ***30.37*** | ***25.09*** | |
|  |  |  |  |  |  |  |  |  |  |  |  |  |  |  |  |  |  |  |  | |
| 24 | no epilepsy | 17.39 | 10.24 | 5.21 | 238.47 | n.d. | 0.16 | 7.35 | 7.10 | 21.19 | 17.78 | 6.58 | 1.13 | 8.61 | 0.42 | 15.57 | 15.57 | 27.43 | 20.71 | |
| 25 | no epilepsy | 29.22 | 20.07 | 8.67 | 293.88 | n.d. | 0.15 | 11.67 | 8.67 | 17.70 | 19.17 | 4.96 | 3.30 | 11.20 | 0.30 | 12.86 | 26.72 | 29.48 | 33.34 | |
| 26 | no epilepsy | 26.59 | 19.88 | 7.89 | 276.30 | n.d. | 1.95 | 11.63 | 10.02 | 20.62 | 20.40 | 5.75 | 2.27 | 11.09 | 0.93 | 16.20 | 26.43 | 34.07 | 30.03 | |
| 27 | no epilepsy | 59.70 | 13.27 | 5.28 | 269.33 | n.d. | 0.18 | 9.12 | 9.12 | 19.95 | 17.55 | 7.59 | 2.86 | 11.29 | 0.66 | 17.32 | 21.22 | 23.30 | 27.63 | |
| 28 | no epilepsy | 25.03 | 16.30 | 7.34 | 267.28 | 1.47 | 1.01 | 9.55 | 14.91 | 18.46 | 23.50 | 5.76 | 1.67 | 8.95 | 0.86 | 14.81 | 19.96 | 30.03 | 20.55 | |
| 29 | no epilepsy | 34.05 | 18.00 | 6.50 | 271.83 | n.d. | 0.11 | 11.82 | 8.02 | 18.63 | 17.52 | 5.50 | 3.42 | 8.97 | 0.20 | 13.38 | 22.96 | 27.72 | 29.23 | |
| 30 | no epilepsy | 14.02 | 14.01 | 3.23 | 206.27 | n.d. | 0.11 | 8.72 | 7.74 | 7.41 | 18.82 | 1.70 | 1.56 | 4.75 | 0.01 | 5.97 | 11.33 | 26.57 | 12.82 | |
| 31 | no epilepsy | 26.18 | 30.19 | 8.22 | 264.45 | n.d. | 0.29 | 22.68 | 5.97 | 17.84 | 21.81 | 5.74 | 2.04 | 11.69 | 2.19 | 13.71 | 18.55 | 49.56 | 42.59 | |
| 32 | no epilepsy | 16.79 | 12.65 | 4.07 | 207.56 | n.d. | 0.25 | 8.19 | 6.29 | 10.65 | 17.93 | 2.15 | 0.88 | 5.43 | 0.66 | 6.72 | 16.22 | 24.46 | 17.99 | |
| 33 | no epilepsy | 29.64 | 9.02 | 6.36 | 303.02 | 0.43 | 0.94 | 18.24 | 12.23 | 16.26 | 23.08 | 4.04 | 2.00 | 93.61 | 0.31 | 11.35 | 13.67 | 27.43 | 18.17 | |
| 34 | no epilepsy | 17.73 | 11.46 | 3.49 | 203.16 | 0.29 | 0.58 | 7.96 | 9.06 | 11.85 | 20.15 | 3.02 | 0.58 | 6.03 | 0.18 | 8.18 | 13.52 | 24.43 | 17.72 | |
| 35 | no epilepsy | 20.77 | 14.03 | 4.83 | 275.86 | n.d. | 0.62 | 9.71 | 10.69 | 13.15 | 18.05 | 4.16 | 2.46 | 7.26 | n.d. | 9.54 | 14.65 | 35.82 | 24.55 | |
| 36 | no epilepsy | 22.14 | 16.51 | 6.18 | 261.99 | 1.41 | 0.47 | 11.12 | 7.91 | 15.70 | 22.31 | 4.89 | 1.12 | 8.89 | 0.55 | 14.00 | 16.12 | 41.43 | 15.00 | |
| 37 | no epilepsy | 24.80 | 17.41 | 6.65 | 269.46 | n.d. | 0.54 | 10.98 | 12.53 | 24.61 | 17.24 | 7.62 | 2.51 | 10.49 | 0.19 | 19.34 | 25.88 | 33.25 | 22.09 | |
| 38 | no epilepsy | 19.04 | 14.47 | 5.40 | 267.55 | n.d. | 0.28 | 9.76 | 8.48 | 12.00 | 17.69 | 4.47 | 1.74 | 8.95 | n.d. | 12.74 | 21.07 | 25.27 | 29.62 | |
| 39 | no epilepsy | 11.82 | 8.57 | 3.16 | 191.74 | n.d. | 0.04 | 6.31 | 6.95 | 12.84 | 16.11 | 2.89 | 0.81 | 6.82 | 0.02 | 7.80 | 12.62 | 23.30 | 12.22 | |
| 40 | no epilepsy | 20.41 | 15.74 | 5.69 | 3.04 | n.d. | 0.28 | 9.01 | 7.17 | 11.82 | 15.63 | 3.68 | 1.77 | 6.80 | n.d. | 9.16 | 15.83 | 24.48 | 12.63 | |
| 41 | no epilepsy | 22.51 | 13.64 | 3.98 | 241.03 | n.d. | 0.22 | 8.23 | 4.96 | 15.27 | 16.66 | 4.87 | 2.19 | 5.86 | n.d. | 11.36 | 19.00 | 18.88 | 22.99 | |
| 42 | no epilepsy | 24.83 | 11.21 | 7.63 | 258.37 | n.d. | 0.12 | 7.65 | 7.31 | 17.31 | 19.19 | 7.49 | 1.53 | 6.60 | 0.51 | 16.11 | 20.06 | 34.22 | 19.05 | |
| 43 | no epilepsy | 32.69 | 20.20 | 9.58 | 313.75 | n.d. | 1.50 | 7.59 | 9.78 | 27.06 | 24.74 | 16.53 | 5.55 | 11.90 | 1.73 | 29.46 | 31.69 | 28.60 | 38.71 | |
| mean |  | ***24.77*** | ***15.34*** | ***5.97*** | ***244.22*** | ***0.90*** | ***0.49*** | ***10.36*** | ***8.75*** | ***16.52*** | ***19.27*** | ***5.47*** | ***2.07*** | ***12.76*** | ***0.61*** | ***13.28*** | ***19.15*** | ***29.49*** | ***23.38*** | |
| SD |  | ***9.87*** | ***4.81*** | ***1.82*** | ***64.13*** | ***0.54*** | ***0.49*** | ***3.76*** | ***2.37*** | ***4.73*** | ***2.53*** | ***3.03*** | ***1.10*** | ***18.68*** | ***0.58*** | ***5.15*** | ***5.31*** | ***6.88*** | ***8.32*** | |
| median |  | ***23.66*** | ***14.25*** | ***5.94*** | ***265.87*** | ***0.92*** | ***0.28*** | ***9.34*** | ***8.25*** | ***16.78*** | ***18.44*** | ***4.93*** | ***1.88*** | ***8.92*** | ***0.46*** | ***13.12*** | ***18.78*** | ***27.58*** | ***21.40*** | |

Mean with standard deviation (SD) and median was calculated for the different clinical groups (focal epilepsy, generalized epilepsy, unclear epilepsy, no epilepsy).
* = patients that received valproate
n.d. = compounds that could not be determined due to low detection (below baseline), a very poor signal-to-noise ratio or strong overlap with an unknown co-eluting peak.

**Table 5D: AA concentrations of the 43 individuals in plasma (pmol/µl)**

| patient | epilepsy | Ala | Arg | Asn | Gln | Asp | Glu | His | Tyr | Val | Gly | Ile | Met | Phe | Pro | Leu | Lys | Ser | Thr |
| --- | --- | --- | --- | --- | --- | --- | --- | --- | --- | --- | --- | --- | --- | --- | --- | --- | --- | --- | --- |
| 1 | focal | 363.40 | 93.70 | 56.39 | 634.29 | 5.68 | 61.27 | 70.22 | 79.96 | 242.32 | 289.74 | 73.97 | 26.19 | 59.75 | 217.19 | 129.21 | 146.95 | 157.26 | 123.82 |
| 2 | focal | 468.09 | 53.72 | 102.09 | 777.90 | 14.48 | 77.40 | 144.43 | 162.17 | 434.83 | 288.24 | 158.28 | 57.43 | 561.07 | 547.54 | 278.08 | 232.27 | 159.83 | 155.74 |
| 3 | focal | 162.29 | 35.62 | 30.40 | 544.61 | 6.18 | 61.61 | 62.80 | 54.41 | 230.76 | 234.10 | 87.37 | 15.50 | 72.00 | 103.78 | 138.93 | 76.63 | 73.56 | 51.75 |
| 4* | focal | 316.03 | 45.09 | 37.23 | 443.06 | 7.03 | 135.32 | 72.77 | 51.00 | 241.07 | 308.69 | 62.24 | 18.21 | 52.67 | 249.78 | 124.39 | 131.49 | 126.70 | 80.13 |
| mean |  | ***327.45*** | ***57.03*** | ***56.53*** | ***599.97*** | ***8.34*** | ***83.90*** | ***87.56*** | ***86.89*** | ***287.25*** | ***280.19*** | ***95.46*** | ***29.33*** | ***186.37*** | ***279.57*** | ***167.65*** | ***146.83*** | ***129.33*** | ***102.86*** |
| SD |  | ***110.09*** | ***22.12*** | ***27.98*** | ***123.01*** | ***3.58*** | ***30.39*** | ***33.04*** | ***44.88*** | ***85.33*** | ***27.81*** | ***37.34*** | ***16.69*** | ***216.44*** | ***163.93*** | ***63.97*** | ***55.82*** | ***34.74*** | ***39.89*** |
| median |  | ***339.72*** | ***49.41*** | ***46.81*** | ***589.45*** | ***6.61*** | ***69.51*** | ***71.50*** | ***67.18*** | ***241.70*** | ***288.99*** | ***80.67*** | ***22.20*** | ***65.88*** | ***233.49*** | ***134.07*** | ***139.22*** | ***141.98*** | ***101.98*** |
|  |  |  |  |  |  |  |  |  |  |  |  |  |  |  |  |  |  |  |  |
| 5 | generalized | 295.11 | 74.49 | 47.10 | 666.58 | 9.95 | 121.08 | 87.52 | 53.07 | 255.43 | 258.61 | 103.97 | 25.36 | 79.16 | 208.39 | 163.85 | 144.98 | 141.09 | 95.83 |
| 6 | generalized | 360.81 | 71.02 | 51.34 | 709.92 | 10.37 | 153.75 | 71.58 | 110.01 | 192.66 | 291.12 | 71.05 | 39.25 | 61.45 | 224.07 | 136.05 | 171.30 | 194.91 | 183.45 |
| 7 | generalized | 280.08 | 78.33 | 59.74 | 598.92 | 9.88 | 1.61 | 81.06 | 46.03 | 276.45 | 354.99 | 84.96 | 25.62 | 57.87 | 262.49 | 150.16 | 182.80 | 159.53 | 176.72 |
| 8 | generalized | 369.12 | 50.41 | 46.04 | 480.73 | 8.42 | 85.19 | 58.29 | 49.77 | 224.93 | 270.55 | 72.99 | 27.44 | 68.17 | 129.49 | 134.15 | 130.58 | 125.87 | 131.79 |
| 9 | generalized | 248.06 | 65.07 | 40.43 | 689.33 | 5.00 | 75.00 | 44.27 | 54.31 | 103.57 | 285.62 | 31.28 | 21.50 | 36.53 | 126.52 | 55.64 | 107.20 | 158.89 | 116.51 |
| 10 | generalized | 334.31 | 74.25 | 56.11 | 673.20 | 31.96 | 127.88 | 90.84 | 66.33 | 226.84 | 555.30 | 97.11 | 31.62 | 60.23 | 268.76 | 154.24 | 188.55 | 224.66 | 185.03 |
| 11 | generalized | 216.92 | 53.93 | 52.30 | 541.13 | 21.49 | 68.01 | 52.60 | 57.85 | 220.46 | 389.83 | 73.41 | 25.03 | 56.34 | 190.49 | 139.54 | 121.81 | 169.52 | 116.84 |
| 12* | generalized | 234.52 | 56.31 | 37.72 | 434.42 | 7.34 | 101.95 | 68.54 | 72.35 | 242.01 | 256.16 | 79.99 | 22.90 | 58.31 | 153.89 | 133.91 | 104.77 | 143.55 | 87.52 |
| 13 | generalized | 611.33 | 164.60 | 95.21 | 804.91 | 41.68 | 191.60 | 89.19 | 96.17 | 263.37 | 441.38 | 88.91 | 0.99 | 114.03 | 340.95 | 161.93 | 289.84 | 270.11 | 235.26 |
| 14 | generalized | 520.73 | 90.77 | 92.10 | 682.12 | 7.92 | 78.11 | 77.95 | 78.88 | 216.89 | 7.33 | 76.09 | 0.72 | 84.54 | 348.68 | 123.32 | 218.54 | 215.64 | 245.19 |
| mean |  | ***347.10*** | ***77.92*** | ***57.81*** | ***628.13*** | ***15.40*** | ***100.42*** | ***72.18*** | ***68.48*** | ***222.26*** | ***311.09*** | ***77.98*** | ***22.04*** | ***67.66*** | ***225.37*** | ***135.28*** | ***166.04*** | ***180.38*** | ***157.41*** |
| SD |  | ***121.42*** | ***31.17*** | ***19.01*** | ***107.60*** | ***11.68*** | ***49.53*** | ***15.34*** | ***20.06*** | ***45.98*** | ***136.19*** | ***18.69*** | ***11.61*** | ***19.85*** | ***75.77*** | ***29.35*** | ***54.56*** | ***42.84*** | ***53.13*** |
| median |  | ***314.71*** | ***72.63*** | ***51.82*** | ***669.89*** | ***9.92*** | ***93.57*** | ***74.76*** | ***62.09*** | ***225.88*** | ***288.37*** | ***78.04*** | ***25.19*** | ***60.84*** | ***216.23*** | ***137.80*** | ***158.14*** | ***164.53*** | ***154.26*** |
|  |  |  |  |  |  |  |  |  |  |  |  |  |  |  |  |  |  |  |  |
| 15 | unclear | 272.81 | 104.14 | 67.70 | 841.34 | 10.46 | 64.19 | 83.75 | 74.73 | 179.95 | 314.27 | 50.70 | 35.60 | 67.71 | 199.78 | 90.75 | 167.88 | 184.42 | 147.68 |
| 16 | unclear | 254.95 | 63.63 | 42.95 | 729.92 | 6.70 | 46.89 | 83.48 | 62.58 | 197.66 | 247.36 | 67.75 | 20.25 | 58.09 | 160.83 | 117.96 | 105.06 | 117.34 | 106.21 |
| 17 | unclear | 238.19 | 65.75 | 58.16 | 705.51 | 9.90 | 117.90 | 85.17 | 65.35 | 232.10 | 228.51 | 83.97 | 29.09 | 59.02 | 174.57 | 140.18 | 102.48 | 145.22 | 100.56 |
| 18 | unclear | 305.54 | 97.45 | 60.55 | 547.08 | 11.97 | 136.21 | 84.90 | 123.42 | 263.91 | 271.83 | 85.06 | 36.59 | 70.34 | 265.23 | 164.39 | 236.46 | 143.82 | 181.56 |
| 19 | unclear | 305.21 | 55.24 | 69.92 | 754.40 | 8.77 | 67.08 | 79.31 | 83.89 | 196.12 | 329.39 | 64.03 | 33.76 | 56.02 | 208.69 | 115.50 | 154.75 | 193.59 | 123.40 |
| 20 | unclear | 260.80 | 17.65 | 50.92 | 555.36 | 11.31 | 114.54 | 59.83 | 34.58 | 173.88 | 311.67 | 49.12 | 19.15 | 42.68 | 132.53 | 87.10 | 107.81 | 185.56 | 109.89 |
| 21 | unclear | 439.98 | 105.31 | 42.00 | 608.68 | 6.62 | 59.28 | 102.94 | 75.30 | 332.91 | 359.78 | 98.44 | 52.02 | 89.50 | 342.94 | 166.35 | 205.36 | 197.52 | 242.65 |
| 22 | unclear | 292.70 | 64.34 | 51.41 | 379.05 | 7.66 | 142.10 | 57.85 | 80.62 | 263.13 | 291.87 | 66.13 | 20.23 | 53.22 | 130.37 | 118.26 | 125.54 | 113.02 | 123.76 |
| 23* | unclear | 532.51 | 74.18 | 67.88 | 632.95 | 9.93 | 216.76 | 87.33 | 72.84 | 204.45 | 393.49 | 70.32 | 37.86 | 53.39 | 345.69 | 122.07 | 159.78 | 165.64 | 176.78 |
| mean |  | ***322.52*** | ***71.97*** | ***56.83*** | ***639.36*** | ***9.26*** | ***107.22*** | ***80.51*** | ***74.81*** | ***227.13*** | ***305.35*** | ***70.61*** | ***31.61*** | ***61.11*** | ***217.85*** | ***124.73*** | ***151.68*** | ***160.68*** | ***145.83*** |
| SD |  | ***92.69*** | ***26.24*** | ***10.02*** | ***129.50*** | ***1.83*** | ***51.23*** | ***13.13*** | ***21.96*** | ***48.61*** | ***49.44*** | ***15.24*** | ***10.12*** | ***12.63*** | ***77.96*** | ***26.45*** | ***44.23*** | ***30.41*** | ***44.11*** |
| median |  | ***292.70*** | ***65.75*** | ***58.16*** | ***632.95*** | ***9.90*** | ***114.54*** | ***83.75*** | ***74.73*** | ***204.45*** | ***311.67*** | ***67.75*** | ***33.76*** | ***58.09*** | ***199.78*** | ***118.26*** | ***154.75*** | ***165.64*** | ***123.76*** |
|  |  |  |  |  |  |  |  |  |  |  |  |  |  |  |  |  |  |  |  |
| 24 | no epilepsy | 178.48 | 39.07 | 43.39 | 490.61 | 5.59 | 72.04 | 58.98 | 57.88 | 291.28 | 224.15 | 120.82 | 19.66 | 81.88 | 174.33 | 201.16 | 125.40 | 101.50 | 102.99 |
| 25 | no epilepsy | 281.76 | 94.68 | 62.17 | 765.20 | 4.83 | 58.83 | 79.19 | 51.55 | 192.47 | 327.55 | 56.04 | 26.35 | 53.32 | 181.04 | 102.23 | 188.57 | 164.08 | 142.46 |
| 26 | no epilepsy | 248.45 | 68.27 | 49.25 | 523.12 | 4.66 | 61.69 | 67.77 | 45.89 | 228.79 | 288.62 | 66.25 | 21.46 | 66.05 | 143.07 | 128.37 | 129.08 | 118.18 | 107.88 |
| 27 | no epilepsy | 565.06 | 59.20 | 44.83 | 494.59 | 6.56 | 69.42 | 61.08 | 65.95 | 242.17 | 277.87 | 82.48 | 24.38 | 77.94 | 353.95 | 137.07 | 109.57 | 120.06 | 124.12 |
| 28 | no epilepsy | 263.23 | 36.69 | 62.53 | 442.00 | 6.91 | 64.03 | 57.62 | 111.56 | 269.25 | 241.73 | 86.76 | 26.50 | 63.18 | 201.02 | 159.92 | 116.00 | 119.77 | 105.23 |
| 29 | no epilepsy | 467.00 | 92.29 | 69.32 | 671.55 | 6.97 | 75.54 | 91.59 | 93.22 | 297.63 | 358.50 | 106.51 | 39.50 | 76.89 | 354.74 | 172.96 | 194.10 | 183.19 | 190.39 |
| 30 | no epilepsy | 276.23 | 84.08 | 57.51 | 552.93 | 4.71 | 64.10 | 76.93 | 76.95 | 185.87 | 344.27 | 53.05 | 29.01 | 55.50 | 164.33 | 109.88 | 107.78 | 209.07 | 106.73 |
| 31 | no epilepsy | 195.43 | 46.84 | 43.18 | 385.15 | 7.16 | 84.33 | 76.43 | 21.71 | 115.85 | 322.00 | 37.61 | 18.44 | 44.13 | 329.76 | 77.44 | 55.23 | 146.39 | 117.90 |
| 32 | no epilepsy | 250.27 | 31.28 | 53.57 | 540.05 | 8.60 | 96.23 | 87.75 | 58.00 | 243.37 | 276.61 | 63.27 | 21.42 | 63.08 | 171.52 | 121.67 | 158.89 | 131.91 | 123.54 |
| 33 | no epilepsy | 322.00 | 30.08 | 39.17 | 451.24 | 7.20 | 40.42 | 53.29 | 50.74 | 166.83 | 194.70 | 56.71 | 20.41 | 409.10 | 99.98 | 96.95 | 89.01 | 111.52 | 70.24 |
| 34 | no epilepsy | 187.67 | 46.19 | 41.40 | 565.49 | 6.41 | 87.55 | 73.97 | 56.44 | 244.30 | 214.14 | 67.03 | 17.07 | 63.02 | 131.12 | 128.23 | 78.82 | 109.60 | 88.22 |
| 35 | no epilepsy | 275.33 | 50.73 | 45.34 | 521.89 | 5.16 | 53.60 | 54.89 | 64.27 | 177.28 | 287.64 | 57.15 | 26.57 | 50.17 | 180.62 | 93.74 | 95.01 | 142.23 | 121.46 |
| 36 | no epilepsy | 274.38 | 99.26 | 53.71 | 660.34 | 29.49 | 134.27 | 77.80 | 51.64 | 215.52 | 312.35 | 67.40 | 19.84 | 70.25 | 120.54 | 135.90 | 102.63 | 188.98 | 80.45 |
| 37 | no epilepsy | 387.12 | 136.51 | 73.46 | 510.35 | 58.48 | 138.55 | 73.35 | 99.83 | 315.65 | 298.95 | 94.21 | 29.77 | 125.46 | 244.92 | 189.59 | 172.79 | 211.96 | 121.74 |
| 38 | no epilepsy | 311.99 | 83.22 | 68.64 | 703.52 | 3.98 | 34.76 | 83.65 | 63.45 | 255.87 | 315.85 | 95.28 | 0.88 | 82.03 | 168.57 | 171.59 | 207.76 | 185.14 | 162.27 |
| 39 | no epilepsy | 249.66 | 29.24 | 50.70 | 480.95 | 10.51 | 114.81 | 65.05 | 64.77 | 233.87 | 256.57 | 70.84 | 18.32 | 73.23 | 146.51 | 136.76 | 115.68 | 162.25 | 93.40 |
| 40 | no epilepsy | 285.56 | 62.10 | 51.94 | 526.33 | 7.52 | 58.90 | 73.38 | 59.77 | 212.82 | 295.01 | 71.15 | 24.82 | 61.70 | 136.58 | 116.36 | 115.51 | 147.21 | 84.95 |
| 41 | no epilepsy | 440.48 | 97.11 | 57.32 | 642.79 | 5.25 | 54.69 | 86.73 | 66.52 | 321.35 | 294.49 | 115.99 | 34.97 | 72.49 | 283.22 | 183.68 | 202.12 | 145.05 | 167.41 |
| 42 | no epilepsy | 401.69 | 64.76 | 110.57 | 828.61 | 8.26 | 105.88 | 85.24 | 97.02 | 322.92 | 292.81 | 163.42 | 51.14 | 83.05 | 311.65 | 264.41 | 213.49 | 238.05 | 162.74 |
| 43 | no epilepsy | 447.95 | 126.77 | 92.69 | 610.35 | 12.21 | 95.56 | 79.19 | 134.69 | 449.63 | 369.03 | 254.30 | 1.56 | 130.00 | 357.87 | 341.26 | 374.67 | 199.56 | 260.91 |
| mean |  | ***315.49*** | ***68.92*** | ***58.53*** | ***568.35*** | ***10.52*** | ***78.26*** | ***73.20*** | ***69.59*** | ***249.14*** | ***289.64*** | ***89.31*** | ***23.60*** | ***90.12*** | ***212.77*** | ***153.46*** | ***147.61*** | ***156.79*** | ***126.75*** |
| SD |  | ***100.81*** | ***30.98*** | ***17.44*** | ***110.44*** | ***12.22*** | ***27.98*** | ***11.23*** | ***25.29*** | ***70.70*** | ***45.04*** | ***47.37*** | ***10.92*** | ***76.13*** | ***84.62*** | ***60.99*** | ***69.10*** | ***38.43*** | ***43.66*** |
| median |  | ***278.99*** | ***63.43*** | ***53.64*** | ***533.19*** | ***6.94*** | ***70.73*** | ***75.20*** | ***63.86*** | ***242.77*** | ***293.65*** | ***70.99*** | ***22.92*** | ***71.37*** | ***177.47*** | ***136.33*** | ***120.70*** | ***146.80*** | ***119.68*** |

Mean with standard deviation (SD) and median was calculated for the different clinical groups (focal epilepsy, generalized epilepsy, unclear epilepsy, no epilepsy).
* = patients that received valproate
